# Furanones and Anthranilic Acid Derivatives from the Endophytic Fungus *Dendrothyrium variisporum*

**DOI:** 10.3390/molecules22101674

**Published:** 2017-10-09

**Authors:** Rémy B. Teponno, Sara R. Noumeur, Soleiman E. Helaly, Stephan Hüttel, Daoud Harzallah, Marc Stadler

**Affiliations:** 1 Department of Microbial Drugs, Helmholtz Centre for Infection Research and German Centre for Infection Research (DZIF), partner site Hannover/Braunschweig, Inhoffenstrasse 7, 38124 Braunschweig, Germany; remyteponno@gmail.com (R.B.T.); noumeur.sara@gmail.com (S.R.N.); soleiman.helaly@aswu.edu.eg (S.E.H.); Stephan.Huettel@helmholtz-hzi.de (S.H.); 2 Department of Chemistry, Faculty of Science, University of Dschang, P.O. Box 67, Dschang, Cameroon; 3 Laboratory of Applied Microbiology, Department of Microbiology, Faculty of Natural and Life Sciences, University Sétif 1 Ferhat Abbas, 19000 Sétif, Algeria; harzallahdaoud@univ-setif.dz; 4 Department of Microbiology-Biochemistry, Faculty of Natural and Life Sciences, University of Batna 2, 05000 Batna, Algeria; 5 Department of Chemistry, Faculty of Science, Aswan University, 81528 Aswan, Egypt

**Keywords:** Montagnulaceae, *Dendrothyrium variisporum*, fermentation, furancarboxylic acid derivatives, anthranilic acid derivatives, antimicrobial activity, cytotoxicity

## Abstract

Extracts from an endophytic fungus isolated from the roots of the Algerian plant *Globularia alypum* showed prominent antimicrobial activity in a screening for novel antibiotics. The producer organism was identified as *Dendrothyrium variisporum* by means of morphological studies and molecular phylogenetic methods. Studies on the secondary metabolite production of this strain in various culture media revealed that the major components from shake flasks were massarilactones D (**1**) and H (**2**) as well as two new furanone derivatives for which we propose the trivial names (5*S*)-*cis*-gregatin B (**3**) and graminin D (**4**). Scale-up of the fermentation in a 10 L bioreactor yielded massarilactone D and several further metabolites. Among those were three new anthranilic acid derivatives (**5**–**7**), two known anthranilic acid analogues (**8** and **9**) and three cyclopeptides (**10**–**12**). Their structures were elucidated on the basis of extensive spectroscopic analysis (1D- and 2D-NMR), high-resolution mass spectrometry (HRESIMS), and the application of the modified Mosher’s method. The isolated metabolites were tested for antimicrobial and cytotoxic activities against various bacteria, fungi, and two mammalian cell lines. The new Metabolite **5** and Compound **9** exhibited antimicrobial activity while Compound **9** showed cytotoxicity activity against KB3.1 cells.

## 1. Introduction

Fungi are a rich source of secondary metabolites that may serve as leads for the development of badly needed novel antibiotics and anticancer agents. In particular, certain ecological groups of fungi like the endophytes have recently been proven to yield a plethora of novel metabolites exhibiting a variety of biological activities [1]. During the course of our studies on endophytic strains derived from plants collected in Algeria, we have encountered various interesting organisms that produced new chemical entities, such as the preussilides from *Preussia similis* [2]. The current study deals with the evaluation of the taxonomy and intensive studies on the secondary metabolite production of another interesting strain *Dendrothyrium variisporum*, a coniothyrium-like fungus that was isolated during the same campaign from *Globularia alypum* Linn. (Plantaginaceae). This is the first report describing the secondary metabolites profile of the genus *Dendrothyrium* Verkley, Göker & Stielow, which was described recently as a new coelomycete genus belonging to the family Montagnulaceae [3].

## 2. Results and Discussion

**Figure 1:**
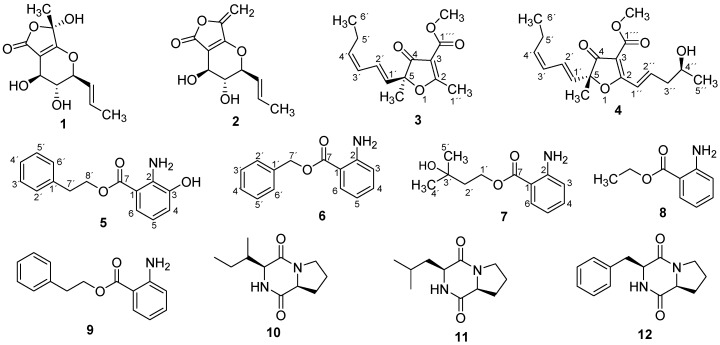
Structures of secondary metabolites isolated from *Dendrothyrium variisporum*.

*Dendrothyrium variisporum* was shown to produce in flasks mainly the polyketide massarilactone D (**1**) and as well as massarilactone H (**2**) and two new minor furanone derivatives **3** and **4**. However, HPLC-MS analysis of the crude extracts revealed the presence of numerous minor constituents that could not be isolated in sufficient quantities to allow for their complete structure elucidation. To increase the amount of biologically active products, a fully controlled bioreactor experiment on a 10 L scale was conducted. A small amount of the culture medium was collected after each 24 h, extracted and analyzed using HPLCDAD-MS for the estimation of metabolite production. After 8 days, the free glucose was completely depleted as determined by commercial glucose test strips, and the cultures were harvested as previously described [4]. From the resulting extracts, Metabolites **5**−**12** were isolated in addition to massarilactone D (Figure 1).

**Figure 2:**
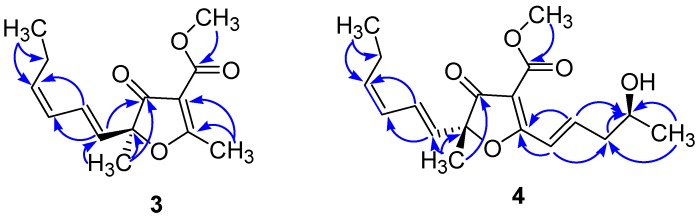
Selected HMBC correlations for Compounds **3** and **4**.

**Figure 3:**
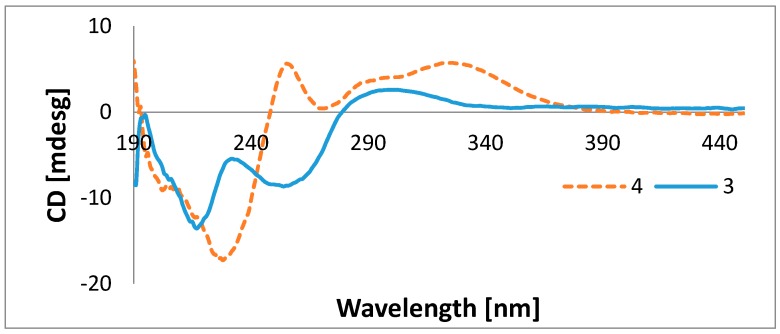
ECD spectra of Compounds **3** and **4** in ethanol.

Compound **3**, isolated as colorless oil, was assigned the molecular formula C_14_H_18_O_4_ (six double-bond equivalents) on the basis of HRESIMS ion cluster [M + H]^+^ at *m*/*z* 251.1276 (Calcd. 251.1278). Its UV spectrum showed intense absorption maxima at 220 and 265 nm. The ^1^H-NMR spectrum exhibited resonances attributed to two tertiary methyl groups at *δ*_H_ 1.56 (s, Me-5) and 2.66 (s, H-1′′), one primary methyl group at *δ*_H_ 1.01 (t, *J* = 7.3 Hz, H-6′), one methoxyl at *δ*_H_ 3.88 (s), and four olefinic proton signals at *δ*_H_ 6.59 (ddd, *J* = 15.5 Hz, 11.2 Hz, 1.3 Hz, H-2′), 5.90 (t, *J* = 11.2 Hz, H-3′), 5.63 (d, *J* = 15.5 Hz, H-1′), and 5.53 (dt, *J* = 10.8 Hz, 7.7 Hz, H-4′) (Table 1). The ^13^C-NMR showed the characteristic signals of furancarboxylic acid derivatives at *δ*_C_ 197.8 (C-4), 195.6 (C-2), 163.4 (C-1′′′), 106.8 (C-3), and 91.4 (C-5) [5,6]. Other important signals were those of olefinic carbons at *δ*_C_ 136.9 (C-4′), 127.6 (C-1′), 126.5 (C-2′), and 126.2 (C-3′). The *E* geometry was assigned to the Δ^1′,2′^ double bond based on the existence of a coupling constant of 15.5 Hz between H-1′ and H-2′, whereas a coupling constant of 10.8 Hz between H-3′ and H-4′ was in favor of the *Z* configuration for the Δ^3′,4′^ double bond [5]. Careful examination of the ^1^H-^1^H COSY, HSQC, and HMBC spectra (Figure 2) proved that **3** was similar to *cis*-gregatin B recently isolated from the ascomycete *Pulvinula* sp. 11120 [6]. The configuration at C-5 was proposed to be *S* based on the negative optical rotation of **3** (−36.67) by opposition to that of (*R*)-*cis*-gregatin B (+168). Furthermore, the negative Cotton effect observed in the 255–265 nm region of the electronic circular dichroism (ECD) curves of Compound **3** in EtOH (Figure 3), when compared to the ECD spectra of aspertetronin A, was indicative of the *S* configuration [7]. The new natural furanone was finally elucidated as methyl (5*S*)-5-[(1′*E*,3′*Z*)-hexa-1,3-dienyl]-5-methyl-4-oxo-2-methyl 4,5-dihydrofuran-3 carboxylate ((5*S*) *cis*-gregatin B).

**Figure 4:**
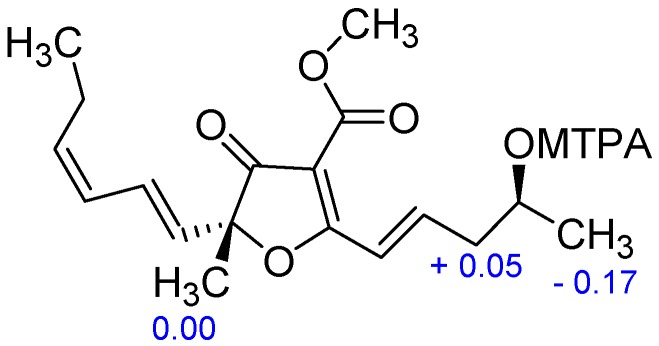
Δ*δ^SR^* values (ppm) for the C-4′′ MTPA esters of Compound **4**.

The molecular formula of Metabolite **4**, also isolated as colorless oil, was deduced to be C_18_H_24_O_5_ from the HRESIMS which showed ion clusters [M + H]^+^ at *m*/*z* 321.1690 (Calcd. 321.1697) and [2M + Na]^+^ at *m*/*z* 663.3130 (Calcd. 663.3140). The UV spectrum showed in addition to the absorption maxima at 216 and 255 nm as in Compound **3**, another band at 310 nm indicative of an extensive conjugated system. The ^1^H-NMR spectrum showed signals attributed to a tertiary methyl group at *δ*_H_ 1.58 (s, Me-5), a secondary methyl group at *δ*_H_ 1.31 (d, *J* = 6.2 Hz, H-5′′), a primary methyl group at *δ*_H_ 1.00 (t, *J* = 7.3 Hz, H-6′), an oxymethine proton at *δ*_H_ 4.08 (m, H-4′′) and one methoxyl (*δ*_H_ 3.88 (s)). The resonances of six olefinic protons at *δ*_H_ 7.41 (dt, *J* = 15.9 Hz, 1.7 Hz, H-1′′), 7.21 (dt, *J* = 15.9 Hz, 7.3 Hz, H-2′′), 6.59 (ddd, *J* = 15.5 Hz, 11.2 Hz, 1.3 Hz, H-2′), 5.90 (t, *J* = 11.2 Hz, H-3′), 5.65 (d, *J* = 15.5 Hz, H-1′), and 5.52 (dt, *J* = 10.8 Hz, 7.7 Hz, H-4′) were also observed (Table 1). Its ^13^C-NMR spectrum exhibited the characteristic signals of furancarboxylic acid derivatives at *δ*_C_ 198.2 (C-4), 184.3 (C-2), 163.4 (C-1′′′), 104.3 (C-3), and 90.7 (C-5). The downfield shift of C-2 (*δ*_C_ 184.3) in Compound **4,** compared to **3** (*δ*_C_ 195.6) was in favor of the fixation of an olefinic carbon at C-2, which was evidenced by carbon signals at *δ*_C_ 121.6 (C-1′′) and 145.1 (C-2′′). This was further confirmed by the HMBC correlation observed between H-1′′ (*δ*_H_ 7.41) and C-2 and between H-2′′ (*δ*_H_ 7.21) and C-2. The ^1^H- and ^13^C-NMR data of **4** were almost identical with those of graminin C [6] with the same chain attached to C-5. The only difference was the presence of one hydroxyl in the fragment attached to C-2. This hydroxyl group was located at C-4′′ as evidenced not only by the ^1^H-^1^H COSY correlation depicted between the secondary methyl signal at *δ*_H_ 1.31 (d, *J* = 6.2 Hz, H-5′′) and the proton at *δ*_H_ 4.08 (m, H-4′′), but also by HMBC correlations observed between H-3′′ (*δ*_H_ 2.55 (m)) and C-4′′ (*δ*_C_ 67.0) and between H-2′′ (*δ*_H_ 7.21 (dt, *J* = 15.9 Hz, 7.3 Hz)) and C-4′′ (*δ*_C_ 67.0) (Figure 2). The *E* geometry was assigned to the Δ^1′,2′^ double bond based on the existence of a coupling constant of 15.5 Hz between H-1′ and H-2′, whereas a coupling constant of 10.8 Hz between H-3′ and H-4′ was in favor of the *Z* configuration for the Δ^3′,4′^ double bond. The geometry of the Δ^1′′,2′′^ double bond was determined from the coupling constant *J*_1′′,2′′_ = 15.9 Hz, indicating an *E*-configuration. The configuration at C-5 was assigned by comparison of its CD characteristics (Figure 3) with those of Compound **3**. The CD spectrum of **4** showed a positive Cotton effect in the 255−265 nm region (opposite to that of **3**), so a 5*R* configuration was concluded. The *S* configuration was assigned to C-4′′ based on the analysis of the Δ*δ^SR^* values of the MTPA esters prepared from **4** [8]. The Δ*δ^SR^* differences of the MTPA esters revealed a negative value of −0.17 for H-5′′, while the Δδ*^SR^* value for H-3′′ was +0.05 ppm (Figure 4) (Table S1). The structure of 4 was finally concluded as (5*R*)-5-[(1′*E*,3′*Z*)-hexa-1,3-dienyl]-5-methyl-4-oxo-2-[(4*S*,1*E*)-4-hydroxypent-1-enyl]-4,5-dihydrofuran-3 carboxylate, trivially named graminin D in agreement with the known compound graminin C without the hydroxyl group at C-4′′[6]. Compounds **3** and **4** are furancarboxylic acid derivatives, a structural class of natural oxygenated heterocycles that have been reported only from fungi of the genera *Aspergillus*, *Cephalosporium* (*Acremonium*), *Paraconiothyrium*, *Penicillium*, and *Pulvinula* [5,6]. Their core structure after several revisions was finally elucidated to 4-(methoxycarbonyl)furan-3(2*H*)-one from degradation reactions and total synthesis [9].

**Figure 5:**
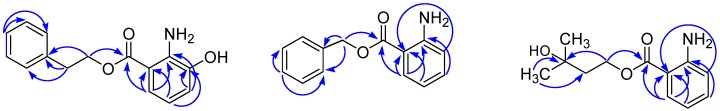
Selected HMBC correlations for Compounds **5**–**7**.

Compound **5** was isolated as a brown gum. Its molecular formula C_15_H_15_NO_3_ was established by the positive ion mode HRESIMS, which showed ion clusters at *m*/*z* 258.1128 [M + H]^+^ (calcd. for C_15_H_16_NO_3_^+^: 258.1125) and 280.0946 [M + Na]^+^ (calcd. for C_15_H_15_NNaO_3_^+^: 280.0944). The absorption maxima observed in the UV spectrum at λ_max_ 226, 260, and 342 nm suggested that 5 might be an anthranilic acid derivative [10]. The ^1^H-NMR spectrum exhibited three signals at *δ*_H_ 7.35 (dd, *J* = 8.1 Hz, 1.4 Hz, H-6), 6.90 (dd, *J* = 7.8 Hz, 1.4 Hz, H-4), and 6.66 (t, *J* = 7.9 Hz, H-5), indicating the presence of a system of three vicinal aromatic protons (Table 3). The two triplets (integrating for two protons each) located at *δ*_H_ 3.06 (t, *J* = 6.9 Hz, H-7′) and 4.48 (t, *J* = 6.9 Hz, H-8′) together with the signal integrating for four protons centered at *δ*_H_ 7.30 (H-2′, H-3′, H-5′ and H-6′) and the multiplet at *δ*_H_ 7.22 (H-4′) were characteristic of a 2-phenylethyl group. The ^13^C-NMR and DEPT NMR spectra showed 15 signals, including those of the 3-hydroxyanthranilic acid moiety at *δ*_C_ 169.2 (C-7), 147.8 (C-3), 137.5 (C-2), 122.8 (C-6), 119.5 (C-4), 119.0 (C-5), and 115.0 (C-1) [11]. The presence of the phenylethyl group was evidenced by resonances of two methylenes at *δ*_C_ 36.3 (C-7′), 66.5 (C-8′), and aromatic carbon signals at *δ*_C_ 139.7 (C-1′), 130.2 (C-2′ and C-6′), 129.7 (C-3′ and C-5′), and 127.7 (C-7′) (Table 2). The gross structure of **5** was determined by a combination of ^1^H–^1^H COSY, HSQC, and HMBC spectra. The correlations shown by the HMBC spectrum (Figure 5) from H-6 (*δ*_H_ 7.35 (dd, *J* = 8.1 Hz, 1.4 Hz)) to C-7 (*δ*_C_ 169.2), C-2 (*δ*_C_ 137.5), and C-1 (*δ*_C_ 115.0) and from H-8′ (*δ*_H_ 4.48 (t, *J* = 6.9 Hz)) to C-7 (*δ*_C_ 169.2), C-7′ (*δ*_C_ 36.3), and C-1′ (*δ*_C_ 139.6) confirmed the structure of the previously unreported Metabolite **5** as 2-phenylethyl 3-hydroxyanthranilate.

Metabolite **6** was also obtained as a brown gum. Its HRESIMS exhibited an ion cluster at *m*/*z* 228.1018 [M + H]^+^ corresponding to the molecular formula C_14_H_13_NO_2_^+^ (calcd. for C_14_H_14_NO_2_^+^: 228.1019). The UV spectrum displayed strong absorptions at 218, 246, and 336 nm. Its ^1^H-NMR spectrum showed four signals at *δ*_H_ 7.83 (dd, *J* = 8.1 Hz, 1.6 Hz, H-6), 7.25 (ddd, *J* = 8.5 Hz, 7.0 Hz, 1.6 Hz, H-4), 6.77 (dd, *J* = 8.3 Hz, 1.0 Hz, H-3), and 6.60 (ddd, *J* = 8.1 Hz, 7.1 Hz, 1.1 Hz, H-5), indicating the presence of a system of four vicinal aromatic protons. The presence of the phenylmethyl (benzyl) moiety instead of a phenylethyl group as in Metabolite **5** was evidenced by a two protons singlet located at *δ*_H_ 5.31 (H-7′) and five aromatic protons at *δ*_H_ 7.44 (dd, *J* = 8.0 Hz, 0.9 Hz, H-2′ and H-6′), 7.38 (td, *J* = 7.1 Hz, 1.6 Hz, H-3′and H-5′), and 7.31 (m, H-4′) (Table 2). Comparison of the ^1^H- and ^13^C-NMR/DEPT data of **6** with those of **5** indicated that the structures of these two compounds are very similar, except the absence of the hydroxyl group at C-3 in **6** and the substitution of the phenylethyl group by the benzyl moiety. This was confirmed by a comprehensive analysis of the 2D-NMR data, particularly ^1^H-^1^H COSY, HSQC, and HMBC spectra. The HMBC correlation depicted between the benzylic proton at *δ*_H_ 5.31 (H-7′) and the carbon at *δ*_C_ 169.3 (C-7) further confirmed the structure (Figure 5). Consequently, Compound **6** was elucidated as phenylmethyl anthranilate. Although it was previously reported as a synthetic compound with a fish anesthetic effect [12], this is the first report on its isolation from the natural source to the best of our knowledge. Furthermore, its NMR data have not yet been reported.

Compound **7** was isolated as a brown gum with the molecular formula C_12_H_17_NO_3_ deduced from the HRESIMS which exhibited ion clusters [M + H]^+^ at *m*/*z* 224.1279 (calcd. for C_12_H_18_NO_3_^+^: 224.1281) and [M + Na]^+^ at 246.1098 (calcd. for C_12_H_17_NO_3_Na^+^: 246.1101). Its UV spectrum showed absorptions at 218, 255, and 334 nm. Detailed analysis of the ^1^H- and ^13^C-NMR spectra, DEPT as well as 2D-NMR data of **7** suggested that the molecule might possess the same anthranilic acid moiety as Compound **6**. This was further supported by the aromatic proton connectivities (H-3/H-4/H-5/H-6) observed in the ^1^H-^1^H COSY spectrum and the HMBC correlations from H-6 (*δ*_H_ 7.81) to C-1 (*δ*_C_ 112.3), C-2 (*δ*_C_ 151.9), and C-7 and from H-3 (*δ*_H_ 6.78) to carbons C-1 (*δ*_C_ 112.3) and C-2 (*δ*_C_ 151.9) (Figure 5). The remaining proton signals were those of two methylenes at *δ*_H_ 1.94 (t, *J* = 7.0 Hz, H-2′) and 4.41 (t, *J* = 7.0 Hz, H-1′) and a singlet integrating for six protons at *δ*_H_ 1.28 (s). The ^13^C-NMR and DEPT spectra of this part revealed signals for two symmetrical methyl groups at *δ*_C_ 29.8 (C-5′ and C-6′), an oxygenated quaternary carbon at *δ*_C_ 70.6 (C-3′), two methylenes including an oxygen-bearing one at *δ*_C_ 62.4 (C-1′) (Table 2). The HMBC correlation from H-4′/H-5′ (*δ*_H_ 1.28) to carbons C-3′ (*δ*_C_ 70.6) and C-2′ (*δ*_C_ 43.0), then from H-1′ (*δ*_H_ 4.41) to C-3′ (*δ*_C_ 70.6) and C-2′ (*δ*_C_ 43.0) confirmed the residue to be 3-hydroxy-3-methylbutyl (Figure 5). Furthermore, the HMBC correlation observed between the oxymethylene protons at *δ*_H_ 4.41 (H-1′) and the anthranilic acid carbonyl proved the structure of **7** to be the previously undescribed 3-hydroxy-3-methylbutyl anthranilate.

From spectroscopic analysis and by comparison with literature data, Compounds **1** and **2** where identified to massarilactone D [13] and massarilactone H [14], respectively. Ethyl anthranilate **8** was previously detected by GC-MS as one of the aroma-active compounds in Pinot Noir wines [15], while 2-phenylethyl anthranilate (**9**) is a fragrance ingredient present in the essential oils from the leaves of *Cinnamomum zeylanicum* Blume collected in India [16]. As far as we know, their ^1^H- and ^13^C-NMR data are reported in the present work for the first time (Table S2). The ^1^H- and ^13^C-NMR data of Compounds **10**–**12** were in agreement with those reported in the literature for cyclo-(l-pro-l-isoleu) [17], cyclo-(L-pro-L-leu) [17,18] and cyclo-(L-pro-L-phe) [18], respectively.

Since the crude extracts showed prominent antimicrobial activity, the isolated metabolites were screened against various bacteria and fungi. The minimum inhibitory concentration (MIC) values showed that only the new Metabolite **5** and Compound **9** were active, whereas the remaining compounds were inactive against the organisms tested (Table 3). Compound **5** showed the strongest activity against *Bacillus subtilis* and *Micrococcus luteus* with MIC values of 8.33 and 16.66 μg/mL, respectively, while the MIC value of Compound **9** against *Mucor hiemalis* (16.66 μg/mL) was the same as that of nystatin used as positive control. The two active metabolites are anthranilic acid derivatives with a phenylethyl core. Since Metabolite **6**, which contains a phenylmethyl group instead of a phenylethyl residue, was not active, it was concluded that the phenylethyl moiety in Compounds **5** and **9** is essential for their antimicrobial activity. Furthermore, the ability of some of the isolated compounds to inhibit the proliferation of two mammalian cell lines including HeLa cells KB3.1 and mouse fibroblasts L929 was examined. Only 2-phenylethyl anthranilate (**9**) showed moderate cytotoxic activity against HeLa cells KB3.1 with an IC_50_ value of 8.8 μg/mL (Table 4).

## 3. Materials and Methods

### 3.1. General Experimental Procedures

Optical rotations were determined with a Perkin Elmer (Überlingen, Germany) 241 MC polarimeter (using the sodium D line and a quartz cuvette with a 10 cm path length and 0.5 mL volume). CD spectra were recorded on a JASCO spectropolarimeter, model J-815 (JASCO, Pfungstadt, Germany). NMR spectra were recorded on a Bruker (Bremen, Germany) 500 MHz Avance III spectrometer with a BBFO (plus) SmartProbe (^1^H 500 MHz, ^13^C 125 MHz) and a Bruker 700 MHz Avance III spectrometer with a 5 mm TCI cryoprobe (^1^H 700 MHz, ^13^C 175 MHz), locked to the deuterium signal of the solvent. Chemical shifts are given in parts per million (ppm), and coupling constants in hertz (Hz). Spectra were measured in methanol-*d*_4_ and deuterated chloroform; chemical shifts were referenced to the solvent signals. HPLCDAD MS analysis was performed using an amaZon speed ETD ion trap mass spectrometer (Bruker Daltonics) in positive and negative ionization modes. The mass spectrometer was coupled to an Agilent 1260 series HPLC-UV system (Agilent Technologies) (Santa Clara, CA, USA) [column 2.1 × 50 mm, 1.7 μm, C18 Acquity uPLC BEH (Waters), solvent A: H_2_O + 0.1% formic acid; solvent B: acetonitrile (ACN) + 0.1% formic acid, gradient: 5% B for 0.5 min, increasing to 100% B in 20 min, maintaining isocratic conditions at 100% B for 10 min, flow = 0.6 mL/min, UV–vis detection 200–600 nm]. HRESIMS spectra were recorded on a maXis ESI TOF mass spectrometer (Bruker Daltonics) [scan range *m*/*z* 100–2500, rate 2 Hz, capillary voltage 4500 V, dry temperature 200 °C], coupled to an Agilent 1200 series HPLC-UV system [column 2.1 × 50 mm, 1.7 μm, C18 Acquity uPLC BEH (Waters), solvent A: H_2_O + 0.1% formic acid; solvent B: ACN + 0.1% formic acid, gradient: 5% B for 0.5 min, increasing to 100% B in 19.5 min, maintaining 100% B for 5 min, FR = 0.6 mL/min, UV–vis detection 200–600 nm]. The molecular formulas were calculated including the isotopic pattern (Smart Formula algorithm). Preparative HPLC purification was performed at room temperature on an Agilent 1100 series preparative HPLC system [ChemStation software (Rev. B.04.03 SP1); binary pump system; column: Kinetex 5u RP C18, dimensions 250 × 21.20 mm; mobile phase: ACN + 0.05% trifluoroacetic acid (TFA) and water + 0.05% TFA; flow rate 20 mL/min; diode-array UV detector; 226 fraction collector].

### 3.2. Fungal Material

The endophytic fungus *Dendrothyrium variisporum* was isolated from fresh healthy roots of *Globularia alypum* Linn. (Plantaginaceae). The plant samples were collected in June 2015 from Ain Touta, Batna 05000 (Algeria). The isolation of the endophyte was achieved following a previously reported method [19,20]. Genomic DNA was extracted from fungal colonies growing on YMG using the EZ-10 Spin Column Genomic DNA Miniprep kit (Bio Basic Canada Inc., Markham, Ontario, Canada) following the manufacturer’s protocol. The internal transcribed spacer (ITS) regions including the 3′-end of 18S rRNA gene, 5.8S rRNA gene, and the 5′-end of the 28S rRNA gene were amplified using ITS1F/ITS4 primer [21]. The ITS sequence of the endophytic fungus was deposited with GenBank under the accession number MG018984. The producer organism was identified as *Dendrothyrium variisporum* on the basis of ITS sequencing and morphology (see Supporting Information).

### 3.3. Fermentation and Extraction

For fermentation in flasks, the submerged culture were raised in 30 × 500 mL Erlenmeyer flasks, each containing 200 mL of YMG medium: 1.0% malt extract, 0.4% glucose, 0.4% yeast extract, pH 6.3 [20]. The flasks were inoculated with five mycelial plugs from actively growing yeast-malt-glucose-agar (YMG) plates and incubated at 23 °C under constant shaking at 140  rpm on a rotary shaker for 12 days. After separation from fungal mycelia by vacuum filtration, the supernatant was treated with 2% (*v*/*v*) of Amberlite XAD-16 resin. The latter was extracted with acetone by sonication at 40 °C for 30 min. The acetone extract was evaporated in vacuo, and the residual aqueous solution was re-extracted with EtOAc*.* The ethyl acetate extract was dried on Na_2_SO_4_ and concentrated by evaporation to yield 1.4 g of crude extract. 

For the scale up, a seed culture of the strain with a total volume of 1000 mL was prepared in YMG medium incubated at 23 °C and 140 rpm for 5 days and homogenized with a Heidolph Silent Crusher. A 15 L in-situ autoclavable bioreactor (bbi Germany) was prepared with 10 L of YMG medium and inoculated with 1000 mL of the seed culture. The temperature was set at 23 °C, agitation with a Rushton-impeller was set to 150 rpm, and the aeration rate was set to 1.5 L/min (0.15 vvm) and remained constant during fermentation. The culture was harvested after 8 days as the glucose was depleted, and a stagnation of secondary metabolite production was observed by analytical HPLC. The mycelium was separated from the culture fluid (supernatant) by centrifugation in a Dupont Instrument (Sorvall RC-5B Refrigerated Superspeed Centrifuge) followed by vacuum filtration and was extracted three times with acetone, then with methanol in an ultrasonic bath at 40 °C for 30 min. The acetone and methanol extracts were filtered, combined and evaporated to yield an aqueous phase, which was further extracted with 3 × 500 mL EtOAc in a separating funnel. The ethyl acetate fraction was dried over anhydrous Na_2_SO_4_, filtered, and concentrated under vacuum to yield 373.7 mg of oily mycelial crude extract. The supernatant was treated with 2% adsorber resin Amberlite XAD-16 resin over 2 h at room temperature. The XAD was separated by filtration and extracted three times with acetone, then with methanol in an ultrasonic bath at 40 °C for 30 min. The extract was evaporated to yield an aqueous phase, which was further extracted with ethyl acetate (3 × 500 mL). After drying over anhydrous Na_2_SO_4_, the ethyl acetate fraction was concentrated under vacuum to yield 508.2 mg of crude extract.

### 3.4. Isolation of Compounds ***1**–**12***

The crude supernatant extract from the fermentation in flasks (858.7 mg) was subjected to flash chromatography (GRACE Reveleris X2 flash system) with silica gel (40 g) as stationary phase. The column was eluted with the mixture of CH_2_Cl_2_ (solvent A) and acetone (solvent B); gradient: 100% A in 5 min, 0–100% B in 35 min, and finally 100% B for 5 min; flow rate: 40 mL/min; UV detection: 254, 280, and 380 nm. Three fractions were collected: Fraction 1 (32.4 mg, *t*_R_ = 8.5−10.1 min), Fraction 2 (32.0 mg, *t*_R_ = 11.5−13.5 min) and Fraction 3 (708.7 mg, *t*_R_ = 14.5−17.1 min), which was pure massarilactone D 1. Fraction 1 was further purified by preparative HPLC using a gradient of 25–60% solvent B in 40 min, 60–100% B for 5 min, and 100% B for 5 min. The fractions were combined according to UV absorption at 220, 280, and 325 nm and concurrent HPLC-MS analyses. Compound **3** (0.4 mg) was eluted at *t*_R_ = 12.80 min, and Compound **4** (1.8 mg) was eluted at *t*_R_ = 9.30 min. Fraction 2 was purified using the same preparative HPLC conditions as for Fraction 1 to yield Compound **2** (9.1 mg) at *t*_R_ = 5.88 min.

The mycelial extract from the bioreactor (373.7 mg) was purified by preparative HPLC (3 runs) using a gradient of 45–80% solvent B in 40 min, 80–100% B for 10 min, and 100% B for 10 min. The fractions were combined according to UV absorption at 220, 240, and 342 nm and concurrent HPLC-MS analyses. Compounds **5** (1.7 mg, *t*_R_ = 11.11 min), **6** (1.6 mg, *t*_R_ = 15.96 min), and **9** (6.1 mg, *t*_R_ = 17.50 min) were obtained. The supernatant extract from the bioreactor (508.2 mg) was submitted to preparative HPLC (4 runs) using a gradient of 15–55% solvent B in 40 min, 55–100% B for 10 min, and 100% B for 10 min (UV absorption at 220, 240, and 342 nm) to yield Compounds **1** (53.6 mg, *t*_R_ = 5.76 min), **7** (3.8 mg, *t*_R_ = 20.87 min), **9** (3.7 mg, *t*_R_ = 44.18 min), **10** (16.4 mg, *t*_R_ = 7.22 min), **11** (24.7 mg, *t*_R_ = 8.12 min), and **12** (7.8 mg, *t_R_* = 9.77 min).

(5*S*) *cis-gregatin B* (**3**): colorless oil; ${\left[\alpha \right]}_{D}^{25}$–36.67 (*c* 0.0006, CHCl_3_); UV (*c* 0.5 mg/mL, EtOH) *λ*_max_ 220, 265 nm; CD (*c* 0.5 mg/mL, EtOH) *λ*_max_ 260 (+) nm; HRESIMS *m*/*z* 251.1276 [M + H]^+^ (calcd. for C_14_H_19_O_4_^+^, 251.1278); ^1^H-NMR (CDCl_3_, 700 MHz) and ^13^C-NMR (CDCl_3_, 175 MHz) data: see Table 1.

*Graminin D* (**4**): colorless oil; ${\left[\alpha \right]}_{D}^{25}$ +49.55 (*c* 0.001, CHCl_3_); UV (*c* 1 mg/mL, EtOH) *λ*_max_ 216, 255, 310 nm; CD (*c* 1 mg/mL, EtOH) *λ*_max_ 260 (–) nm; HRESIMS *m*/*z* 321.1690 [M + H]^+^ (calcd. for C_18_H_25_O_5_^+^, 321.1697); ^1^H-NMR (CDCl_3_, 700 MHz) and ^13^C-NMR (CDCl_3_, 175 MHz) data: see Table 1.

*2-Phenylethyl 3-hydroxyanthranilate* (**5**): brown gum; UV *λ*_max_ 226, 260, 342 nm; HRESIMS *m*/*z* 258.1128 [M + H]^+^ (calcd. for C_15_H_16_ NO_3_^+^, 258.1125), 280.0946 [M + Na]^+^ (calcd. for C_15_H_15_NNaO_3_^+^, 280.0944); ^1^H-NMR (CD_3_OD, 700 MHz) and ^13^C-NMR (CD_3_OD, 175 MHz) data: see Table 2.

*Phenylmethyl anthranilate* (**6**): brown gum; UV *λ*_max_ 218, 246, 336 nm; HRESIMS *m*/*z* 228.1016 [M + H]^+^ (calcd. for C_14_H_14_ NO_2_^+^, 228.1019) ^1^H-NMR (CD_3_OD, 700 MHz) and ^13^C-NMR (CD_3_OD, 175 MHz) data: see Table 2.

*3-Hydroxy-3-methylbutyl anthranilate* (**7**): brown gum; UV *λ*_max_ 218, 255, 334 nm; HRESIMS *m*/*z* 224.1279 [M + H]^+^ (calcd. for C_12_H_18_NO_3_^+^, 224.1281), 246.1098 [M + Na]^+^ (calcd. for C_12_H_17_NaNO_3_^+^, 246.1101); ^1^H-NMR (CD_3_OD, 500 MHz) and ^13^C-NMR (CD_3_OD, 125 MHz) data: see Table 2.

### 3.5. Preparation of the S- and R-MTPA Esters of ***4***

*R*-(–)-MTPA-Cl (4 μL) was added to a stirred solution of **4** (0.2 mg) and dry pyridine (5 μL) in dry CDCl_3_ (100 μL) at room temperature. After 1 h, the reaction mixture was diluted by the addition of 100 μL of CDCl_3_. The produced *S*-MTPA ester of **4** was submitted to NMR spectroscopy. In an entirely analogous fashion, the *R*-MTPA ester of **4** was prepared using *S*-(+)-MTPA-Cl. The ^1^H-NMR data of the *R* and *S* derivatives are presented in the supporting information (Table S2).

### 3.6. Biological Activities

The MIC and the in vitro cytotoxicity (IC_50_) were determined according to our previously reported procedures [2,22,23]. Briefly, minimum inhibitory concentrations (MICs) in μg/mL of the isolated compounds were determined by serial dilution assay against *Schizosaccharomyces pombe* DSM 70572, *Pichia anomala* DSM 6766, *Mucor hiemalis* DSM 2656, *Candida albicans* DSM 1665, *Rhodoturula glutinis* DSM 10134, *Micrococcus luteus* DSM 1790, *Bacillus subtilis* DSM 10, *Escherichia coli* DSM 1116, *Staphylococcus aureus* DSM 346, *Mycobacterium smegmatis* DSM ATCC 700084*, Chromobacterium violaceum* DSM 30191, and *Pseudomonas aeruginosa* DSM PA14. The assays were carried out in 96-well microtiter plates in YMG media for filamentous fungi and yeast and EBS for bacteria. Gentamycin, kanamycin, nystatin, and oxytetracyclin were used as positive control, and the negative control was methanol. The cytotoxicity against HeLa cells KB3.1 and mouse fibroblasts L929 cells was determined by using the MTT (2-(4,5-dimethylthiazol-2-yl)-2,5-diphenyltetrazolium bromide) method in 96-well microplates. The cell lines were cultured in DMEM (Gibco). Briefly, 60 μL aliquots of serial dilutions from an initial stock of 1 mg/mL in MeOH of the test compounds were added to 120 μL aliquots of a cell suspension (5 × 10^4^ cells/mL) in 96-well microplates. After 5 days incubation, an MTT assay was performed, and the absorbance measured at 590 nm using an ELISA plate reader (Victor). The concentration at which the growth of cells was inhibited to 50% of the control (IC_50_) was obtained from the dose–response curves. Epothilone B was used as a positive control, while methanol was used as a negative control.

## 4. Conclusions

During the course of our studies on endophytic strains derived from plants collected in Algeria for the discovery of new antibiotics, 12 metabolites, including five new compounds, were isolated from the fungus *Dendrothyrium variisporum* harbored in *Globularia alypum* Linn. (Plantaginaceae). The anthranilic acid derivatives **5** and **9** exhibited antimicrobial activity, whereas the remaining compounds were inactive against the organisms tested.

## Figures and Tables

**Table 1:** ^13^C- and ^1^H-NMR spectroscopic data of Compounds **3** and **4** (175 and 700 MHz, resp.; CDCl_3_; *δ* in ppm).

Position	3		4	
	*δ*_C_, Type	*δ*_H_ (*J* in Hz)	*δ*_C_, Type	*δ*_H_ (*J* in Hz)
2	195.6, C	/	184.3, C	/
3	106.8, C	/	104.3, C	/
4	197.8, C	/	198.2, C	/
5	91.4, C	/	90.7, C	/
1′	127.6, CH	5.63, d (15.5)	128.2, CH	5.65, d (15.5)
2′	126.5, CH	6.59, ddd (15.5, 11.2, 1.3)	126.5, CH	6.59, ddd (15.5, 11.2, 1.3)
3′	126.2, CH	5.90, t (11.2)	126.3, CH	5.90, t (11.2)
4′	136.9, CH	5.53, d (10.8, 7.7)	136.7, CH	5.52, dt (10.8, 7.7)
5′	21.2, CH_2_	2.21, m	21.1, CH_2_	2.19, m
6′	14.1, CH_3_	1.01, t (7.3)	14.1, CH_3_	1.00, t (7.3)
1′′			121.6, CH	7.41, dt (15.9, 1.7)
2′′			145.1, CH	7.21, dt (15.9, 7.3)
3′′			42.9, CH_2_	2.55, m
4′′			67.0, CH	4.08, m
5′′			23.6, CH_3_	1.31, d (6.2)
1′′′	163.4, C	/	163.4, C	/
Me-5	22.5, CH_3_	1.56, s	22.5, CH_3_	1.58, s
OMe	51.6, CH_3_	3.83, s	51.7, CH_3_	3.85, s

**Table 2:** ^13^C- and ^1^H-NMR spectroscopic data of Compounds **5**–**7** in CD_3_OD.

Position	5 ^a^		6 ^a^		7 ^c^	
	*δ*_C_	*δ*_H_ (*J* in Hz)	*δ*_C_	*δ*_H_ (*J* in Hz)	δ_C_	*δ*_H_ (*J* in Hz)
1	115.0, C	/	111.7, C	/	112.3, C	/
2	137.5, C	/	152.5, C	/	151.9, C	/
3	147.8, C	/	118.2, CH	6.77, dd (8.3, 1.0)	118.4, CH	6.78, dd (8.3, 1.0)
4	119.5, CH	6.90, dd (7.8, 1.4)	135.4, CH	7.25, ddd (8.5, 7.0, 1.6)	135.2, CH	7.26, ddd (8.5, 7.1, 1.6)
5	119.0, CH	6.66, t (7.9)	117.2, CH	6.60, ddd (8.1, 7.1, 1.1)	117.5, CH	6.62, ddd (8.2, 7.1, 1.1)
6	122.8, CH	7.35, dd (8.1, 1.4)	132.2, CH	7.83, dd ( 8.1, 1.6)	132.2, CH	7.81, dd ( 8.1, 1.6)
7	169.2, C	/	169.3, C	/	169.6, C	/
1′	139.7, C	/	138.2, C	/	62.4, CH_2_	4.41, t (7.0)
2′	130.2, CH	7.30, m ^b^	129.2, CH	7.44, dd (8.0, 0.9)	43.0, CH_2_	1.94, t (7.0)
3′	129.7, CH	7.30, m ^b^	129.7, CH	7.38, td (7.1, 1.6)	70.6, C	/
4′	127.7, CH	7.22, m	129.3, CH	7.31, m	29.8, CH_3_	1.28, s
5′	129.7, CH	7.30, m ^b^	129.7, CH	7.38, td (7.1, 1.6)	29.8, CH_3_	1.28, s
6′	130.2, CH	7.30, m ^b^	129.2, CH	7.44, dd (8.0, 0.9)		
7′	36.3, CH_2_	3.06, t (6.9)	67.1, CH_2_	5.31, s		
8′	66.5, CH_2_	4.48, t (6.9)				

^a^ 175 and 700 MHz, resp.; ^b^ Overlapped; ^c^ 125 and 500 MHz, resp.

**Table 3:** MIC [μg/mL] values of Compounds **1**, **2**, **4**–**12** against the tested microorganisms.

Test Organism	MIC (μg/mL)											
	1	2	4	5	6	7	8	9	10	11	12	References
*Schizosaccharomyces* *pombe* DSM 70572	n.a.	n.a.	n.a.	n.a.	n.a.	n.a.	n.a.	n.a.	n.a.	n.a.	n.a.	16.66 ^n^
*Pichia anomala* DSM 6766	n.a.	n.a.	n.a.	n.a.	n.a.	n.a.	n.a.	n.a.	n.a.	n.a.	n.a.	8.33 ^n^
*Mucor hiemalis* DSM 2656	n.a.	n.a.	n.a.	33.33	n.a.	n.a.	n.a.	16.66	n.a.	n.a.	n.a.	16.66 ^n^
*Candida albicans* DSM 1665	n.a.	n.a.	n.a.	n.a.	n.a.	n.a.	n.a.	n.a.	n.a.	n.a.	n.a.	16.66 ^n^
*Rhodoturula glutinis* DSM 10134	n.a.	n.a.	n.a.	66.67	n.a.	n.a.	n.a.	33.33	n.a.	n.a.	n.a.	2.08 ^n^
*Micrococcus luteus* DSM 1790	n.a.	n.a.	n.a.	16.66	n.a.	n.a.	n.a.	n.a.	n.a.	n.a.	n.a.	0.40 ^o^
*Bacillus subtilis* DSM 10	n.a.	n.a.	n.a.	8.33	n.a.	n.a.	n.a.	66.67	n.a.	n.a.	n.a.	4.16 ^o^
*Escherichia* *coli* DSM 1116	n.a.	n.a.	n.a.	n.a.	n.a.	n.a.	n.a.	n.a.	n.a.	n.a.	n.a.	3.33 ^o^
*Staphylococcus* *aureus* DSM 346	n.a.	n.a.	n.a.	66.67	n.a.	n.a.	n.a.	66.67	n.a.	n.a.	n.a.	0.10 ^o^
*Mycobacterium smegmatis* DSM ATCC 700084	n.a.	n.a.	n.a.	n.a.	n.a.	n.a.	n.a.	n.a.	n.a.	n.a.	n.a.	2.08 ^k^
*Chromobacterium violaceum* DSM 30191	n.a.	n.a.	n.a.	n.a.	n.a.	n.a.	n.a.	n.a.	n.a.	n.a.	n.a.	0.40 ^o^
*Pseudomonas aeruginosa* DSM PA14	n.a.	n.a.	n.a.	n.a.	n.a.	n.a.	n.a.	n.a.	n.a.	n.a.	n.a.	0.52 ^g^

n.a.: No Activity, ^g^ Gentamycin 1 mg/mL, ^k^ Kanamycin 10 mg/mL, ^n^ Nystatin 1 mg/mL, ^o^ Oxytetracyclin 1 mg/mL.

**Table 4:** Cytotoxic effect (IC_50_) of Compounds **1**, **2**, and **4**–**12** against two cancer cell lines.

	IC_50_ (μg/mL)											IC_50_ (ng/mL)
Cell Line	1	2	4	5	6	7	8	9	10	11	12	Epothilone B
KB3.1	n.a.	n.a.	18	18	n.a.	n.a.	n.a.	8.8	n.a.	n.a.	n.a.	0.052
L929	n.a.	n.a.	19	no	n.a.	n.a.	n.a.	n.o.	n.a.	n.a.	n.a.	1.4

n.a.: Not active; no: IC_50_ not obtained.

## Supplementary Materials

The description of the producer organism, LC-MS spectra, HRESIMS, ^1^H- and ^13^C-NMR, ^1^H-^1^H COSY, HSQC, and HMBC spectra are available as Supplementary Material.
