# Supplementary material for: Furanones and Anthranilic Acid Derivatives from the Endophytic Fungus Dendrothyrium variisporum
Source: Molecules. 2017 Oct 9;22(10):1674. doi: 10.3390/molecules22101674 (PMC6151570; doi:10.3390/molecules22101674)
Supplement: Supplementary file 1 [file molecules-22-01674-s001.pdf]

Furanones and Anthranilic Acid Derivatives from the Endophytic fungus *Dendrothyrium variisporum*

Rémy B. Teponno[1,2,†], Sara R. Noumeur[1,3,4,†], Soleiman E. Helaly[1,5], Stephan Hüttel[1], Daoud Harzallah[3], and Marc Stadler[1,*]

[1]Department of Microbial Drugs, Helmholtz Centre for Infection Research and German Centre for Infection Research (DZIF), partner site Hannover/Braunschweig, Inhoffenstrasse 7, 38124 Braunschweig, Germany; remyteponno@gmail.com (R.B.T.); noumeur.sara@gmail.com (S.R.N.); soleiman.helaly@aswu.edu.eg (S.E.H.); Stephan.Huettel@helmholtz-hzi.de (S.H.)

[2]Department of Chemistry, Faculty of Science, University of Dschang, P.O. Box 67, Dschang, Cameroon

[3]Laboratory of Applied Microbiology, Department of Microbiology, Faculty of Natural and Life Sciences, University Sétif 1 Ferhat Abbas, 19000 Sétif, Algeria

[4]Department of Microbiology-Biochemistry, Faculty of Natural and Life Sciences, University of Batna 2, 05000 Batna, Algeria

[5]Department of Chemistry, Faculty of Science, Aswan University, 81528 Aswan, Egypt

* Correspondence: marc.stadler@helmholtz-hzi.de ; Tel.: +49 531 6181-4240; Fax: +49 531 6181 9499

[†]These authors contributed equally to this work.

Figure S1. Morphological characteristics of *Dendrothyrium variisporum*.

Figure S2. LC-MS chromatogram of Massarilactone D (**1**)

Figure S3. LC-MS chromatogram of Massarilactone H (**2**)

Figure S4. LC-MS chromatogram of compound **3**

Figure S5. ^{1}H NMR spectrum of compound **3** (700 MHz, CDCl$_3$)

Figure S6. ^{13}C NMR spectrum of compound **3** (175 MHz, CDCl$_3$)

Figure S7. DEPT spectrum of compound **3** (175 MHz, CDCl$_3$)

Figure S8. ^{1}H-^{1}H COSY spectrum of compound **3**

Figure S9. HSQC spectrum of compound **3**

Figure S10. HMBC spectrum of compound **3**

Figure S11. LC-MS chromatogram of compound **4**

Figure S12. ^{1}H NMR spectrum of compound **4** (700 MHz, CDCl$_3$)

Figure S13. ^{13}C NMR spectrum of compound **4** (175 MHz, CDCl$_3$)

Figure S14. DEPT spectrum of compound **4** (175 MHz, CDCl$_3$)

Figure S15. ^{1}H-^{1}H COSY spectrum of compound **4**

Figure S16. HSQC spectrum of compound **4**

Figure S17. HMBC spectrum of compound **4**

Figure S18. LC-MS chromatogram of compound **5**

Figure S19. ^{1}H NMR spectrum of compound **5** (700 MHz, CD$_3$OD)

Figure S20. ^{13}C NMR spectrum of compound **5** (175 MHz, CD$_3$OD)

Figure S21. DEPT spectrum of compound **5** (175 MHz, CD$_3$OD)

Figure S22. ^{1}H-^{1}H COSY spectrum of compound **5**

Figure S23. HMBC spectrum of compound **5**

Figure S24. LC-MS chromatogram of compound **6**

Figure S25. ^{1}H NMR spectrum of compound **6** (700 MHz, CD$_3$OD)

Figure S26. ^{13}C NMR spectrum of compound **6** (175 MHz, CD$_3$OD)

Figure S27. DEPT spectrum of compound **6** (175 MHz, CD$_3$OD)

Figure S28. ^{1}H-^{1}H COSY spectrum of compound **6**

Figure S29. HSQC spectrum of compound **6**

Figure S30. HMBC spectrum of compound **6**

Figure S31. LC-MS chromatogram of compound **7**

Figure S32. ^{1}H NMR spectrum of compound **7** (500 MHz, CD_3OD)

Figure S33. ^{13}C NMR spectrum of compound **7** (125 MHz, CD_3OD)

Figure S34. DEPT spectrum of compound **7** (125 MHz, CD_3OD)

Figure S35. ^{1}H-^{1}H COSY spectrum of compound **7**

Figure S36. HSQC spectrum of compound **7**

Figure S37. HMBC spectrum of compound **7**

Figure S38. LC-MS chromatogram of compound **8**

Figure S39. LC-MS chromatogram of compound **9**

Figure S40. LC-MS chromatogram of compound **10**

Figure S41. LC-MS chromatogram of compound **11**

Figure S42. LC-MS chromatogram of compound **12**

Table S1. ^{1}H-NMR spectroscopic data for (*S*)- and (*R*)-MTPA esters of compound **1** ($CDCl_3$, 700 MHz)

Table S2. ^{13}C- and ^{1}H-NMR spectroscopic data of compounds **8** and **9** (CD_3OD, 125 and 500 MHz, resp.)

Table S3. MIC [µg/mL] values of compounds **1**, **2**, **4-12** sgainst the tested microorganisms

Table S4. Cytotoxic effect (IC_{50}) of compounds **1**, **2**, **4-12** against two cancer cell lines

Strain characterization

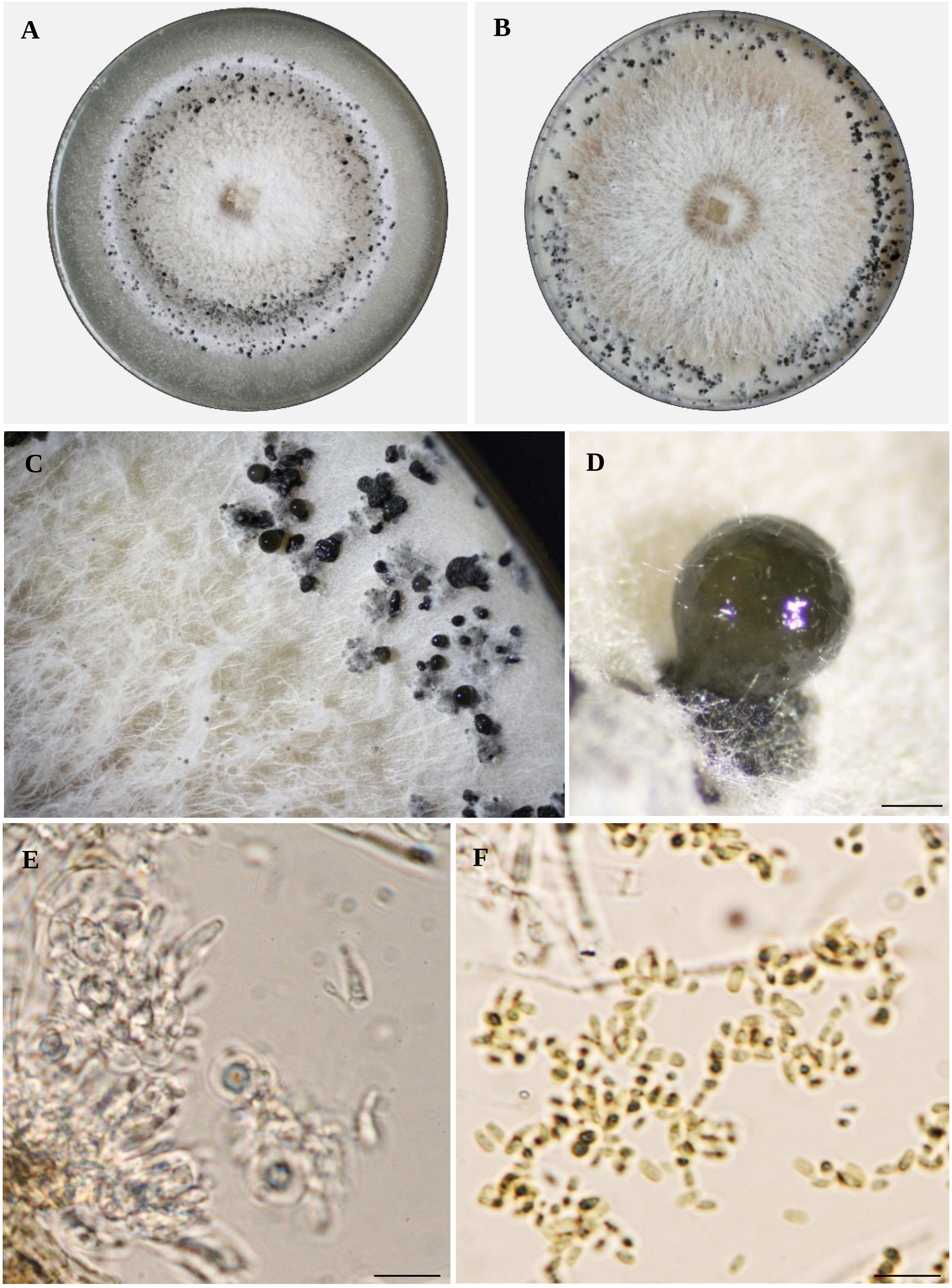

Figure S1. Morphological characteristics of *Dendrothyrium variisporum*. A culture on PDA after 21 days on 9 cm dish, B culture on OMA after 21 days on 9 cm dish , C Conidiomata forming on the edge of culture on OMA medium, D Conidiomata, E Conidiogenous cells, F conidia, scale bar E, F 10 μm, D100 μm.

Morphology and size of macroscopic features of the culture on the plate were determined. Colonies on PDA and OMA spread over the whole 9cm dish after 21 days. Colonies on PDA appear white in the center with an even colourless margin. On OMA, colonies with even coulouress margin

and white dense aerial mycelium in the center are observed. Conidiomata eustromatic, formed on the edge of culture, often merged to complexes, dark brown to dark, reaching 300-500μm of diameter. The outer surface covered by glabrous hyphae. Conidia (1-2μm)×(3-4 μm), aseptate, variable in shape: subglobose, ellipsoid or obovoid, olivceous-brown. Conidiogeous cells (12-20)×(2-3)μm, acropleurogenous condiophore [1].

After Local Alignment Search Tool (BLAST) search of the nucleotide sequence ITS from the National Center for Biotechnology Information database (http://www.ncbi.nlm.nih.gov/), the ITS rDNA sequence of the producer fungus exhibited 100% homology to *Dendrothyrium variisporum* CBS 121517.

Based on both morphological and molecular data, the producer strain could be identified as *Dendrothyrium variisporum*

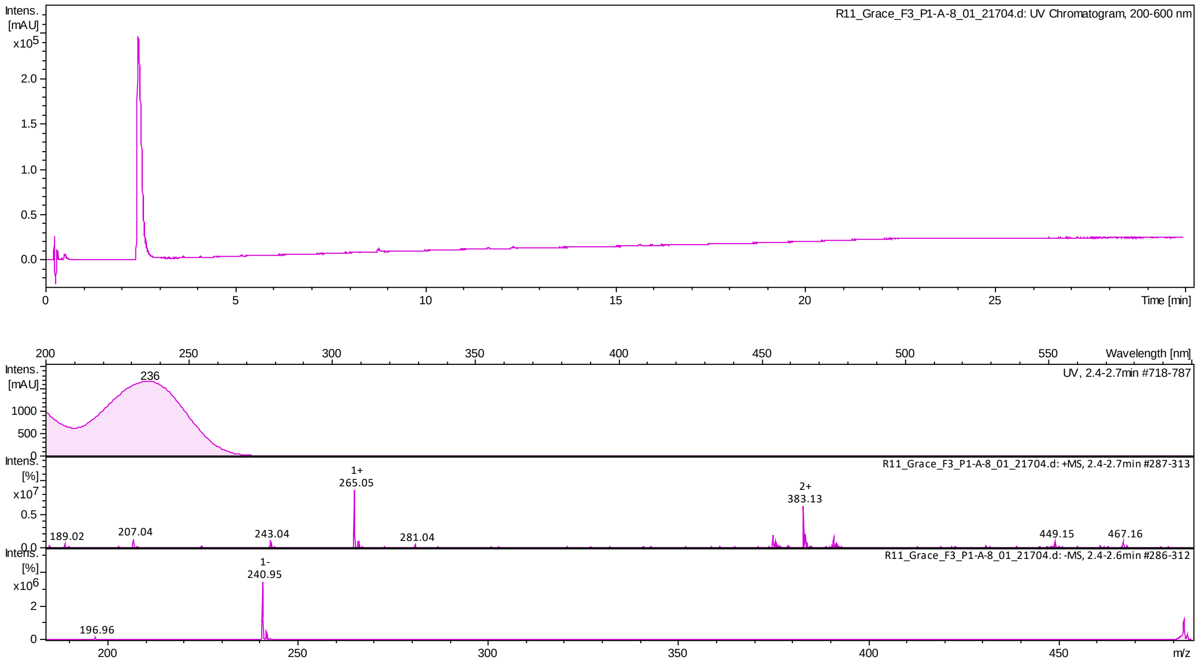

Figure S2. LC-MS chromatogram of Massarilactone D (**1**)

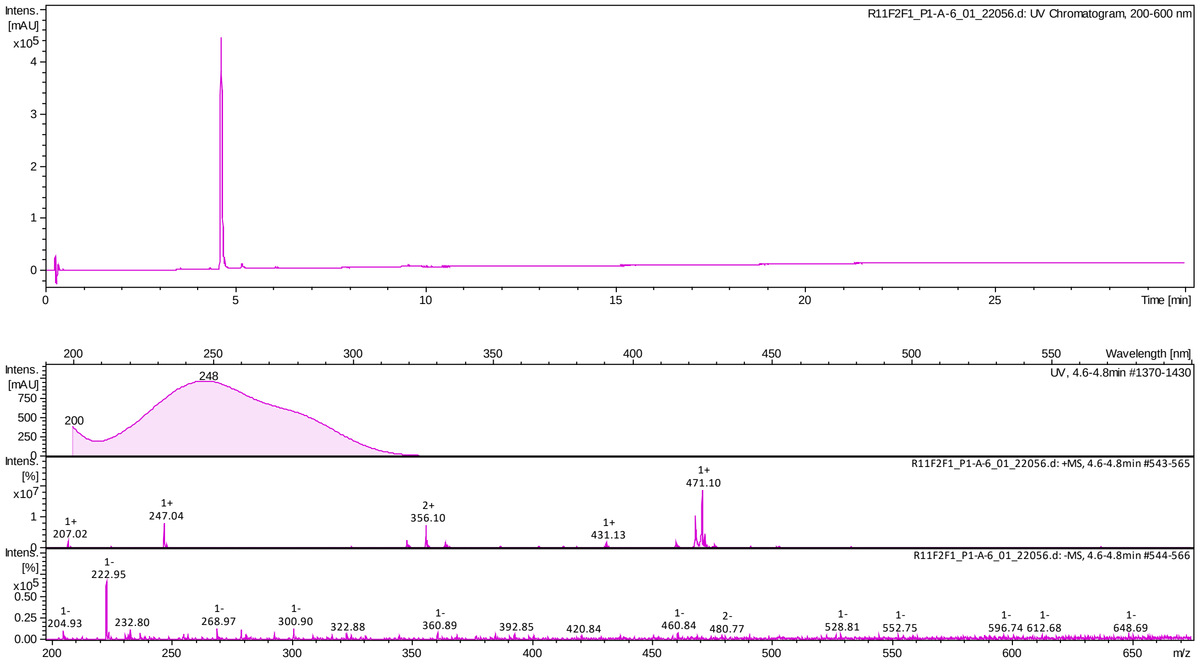

Figure S3. LC-MS chromatogram of Massarilactone H (**2**)

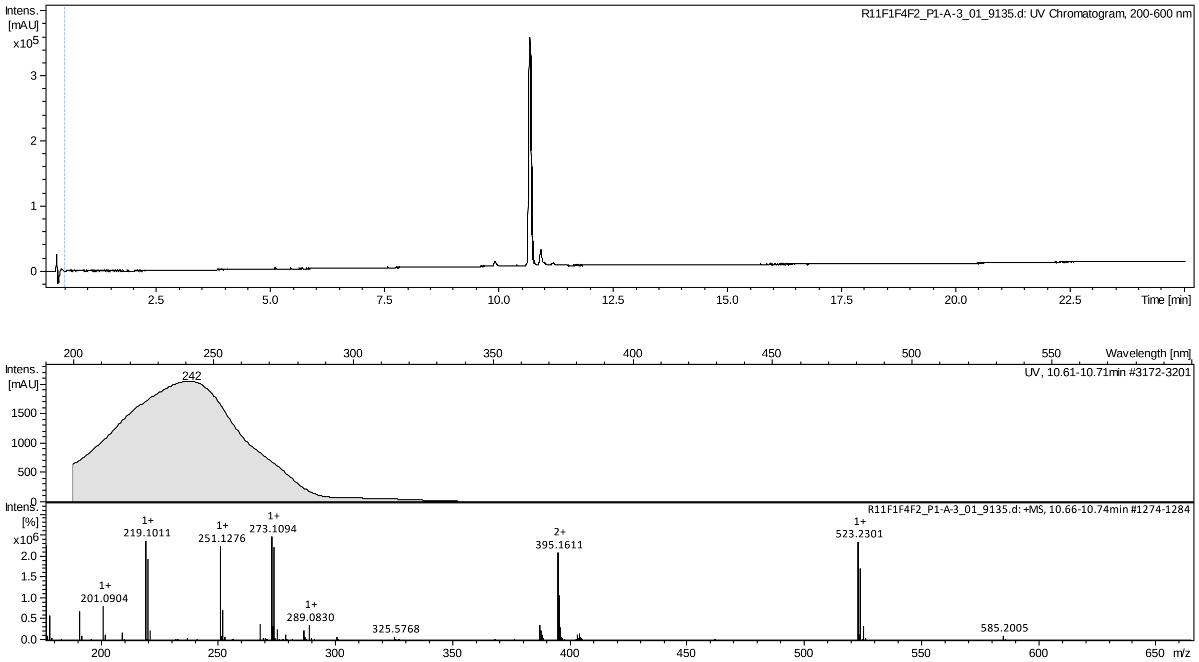

Figure S4. LC-MS chromatogram of compound **3**

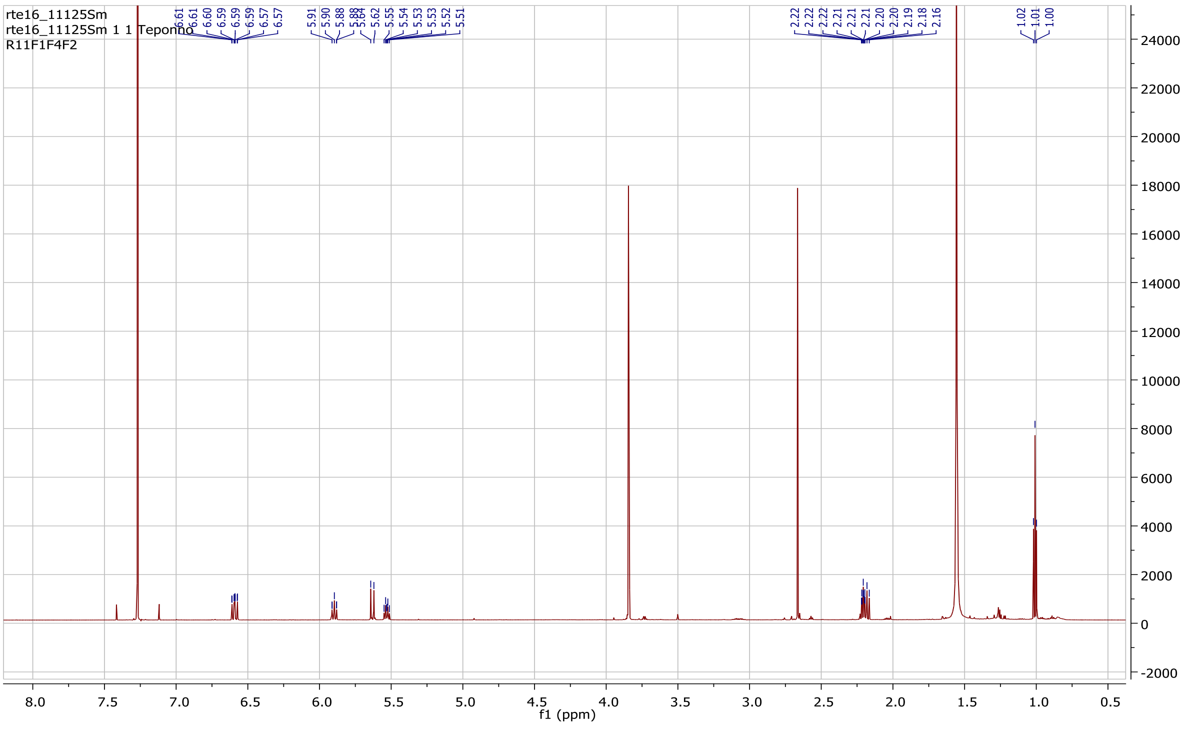

Figure S5. ^{1}H NMR spectrum of compound **3** (700 MHz, $CDCl_3$)

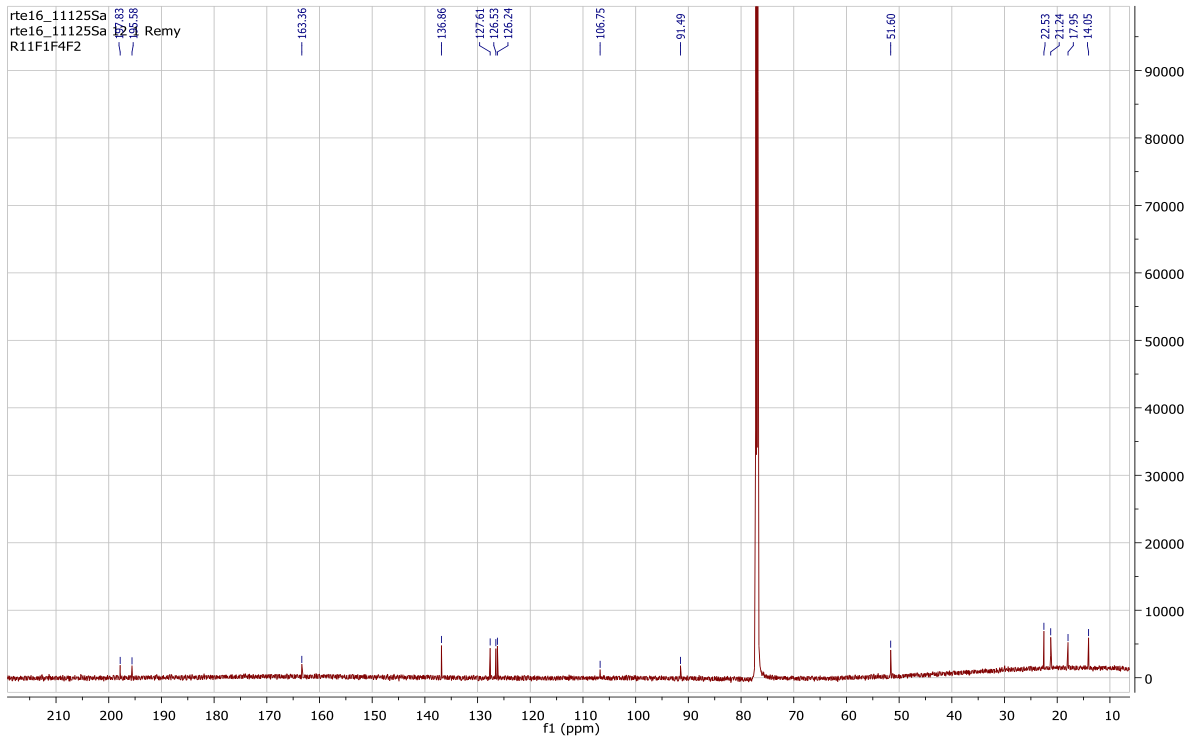

Figure S6. ^{13}C NMR spectrum of compound **3** (175 MHz, $CDCl_3$)

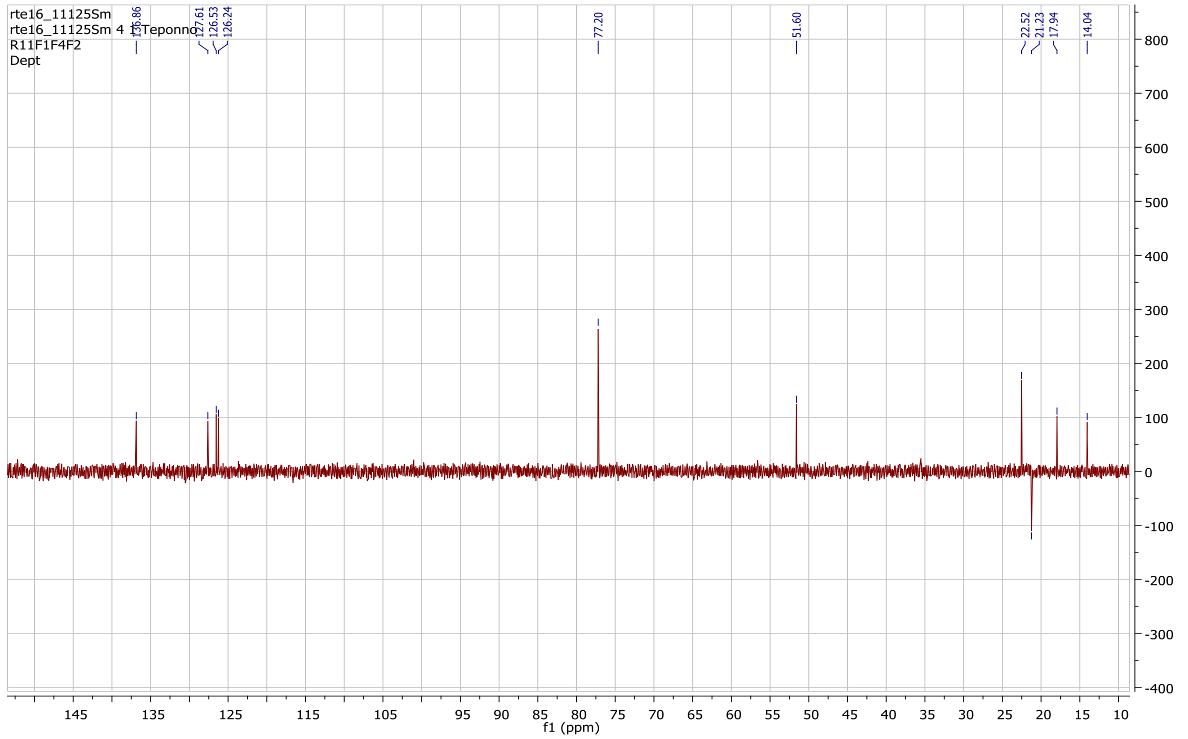

Figure S7. DEPT spectrum of compound **3** (175 MHz, $CDCl_3$)

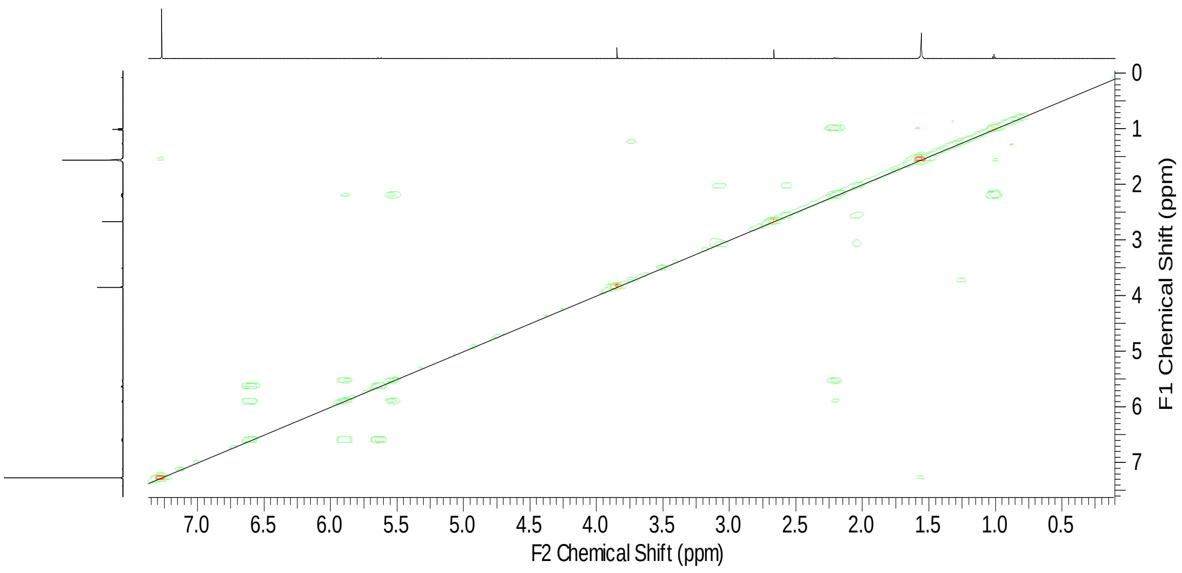

Figure S8. ^{1}H-^{1}H COSY spectrum of compound **3**

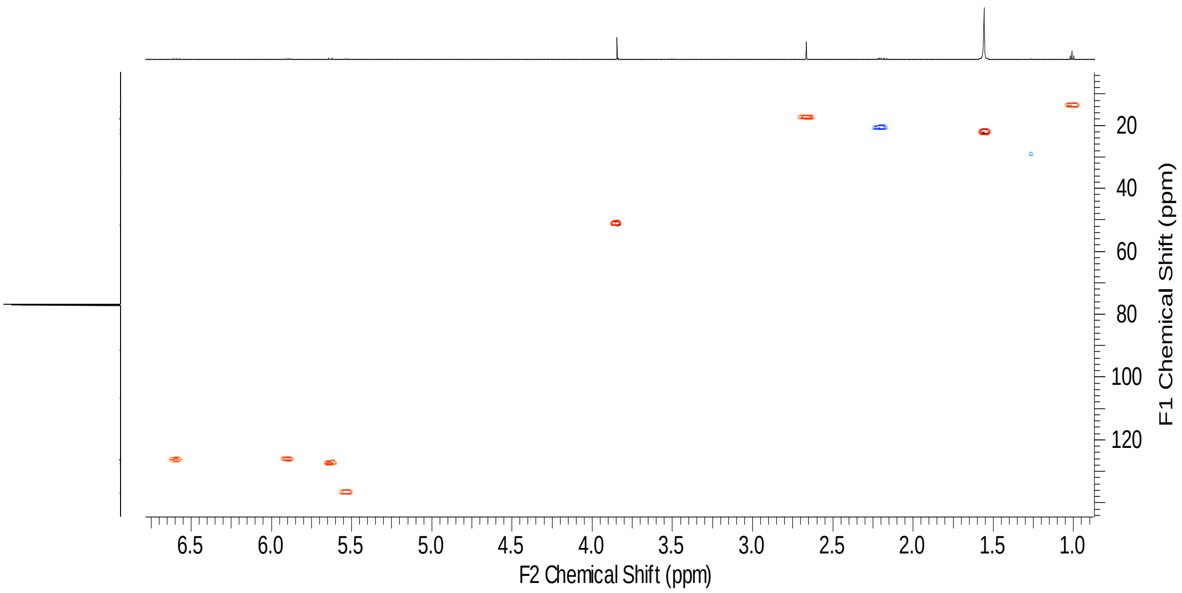

Figure S9. HSQC spectrum of compound **3**

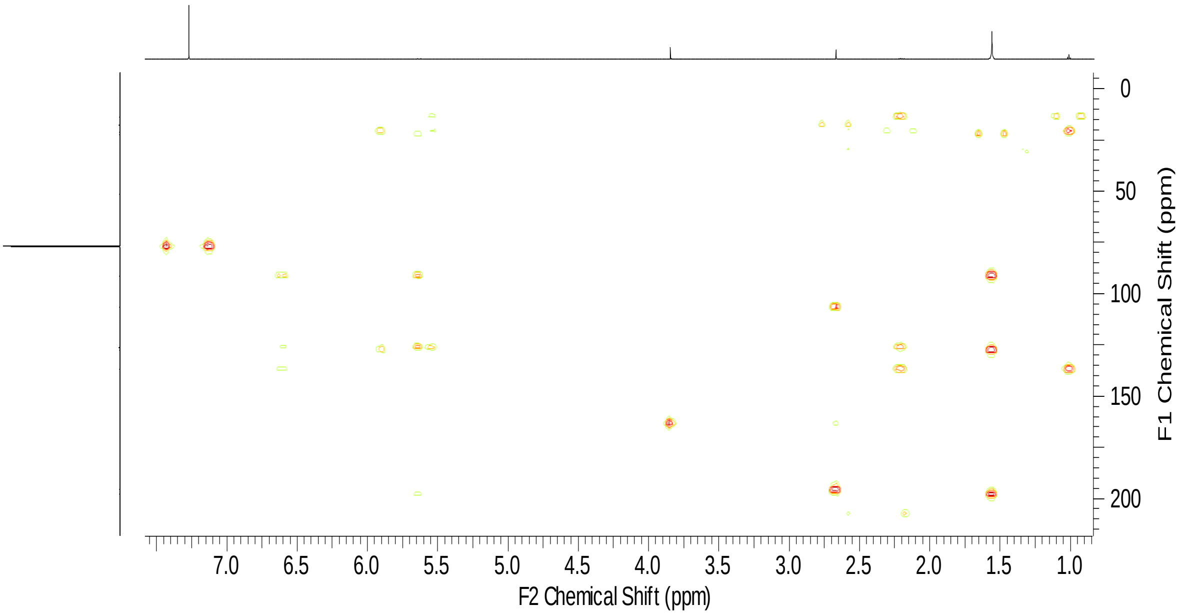

Figure S10. HMBC spectrum of compound **3**

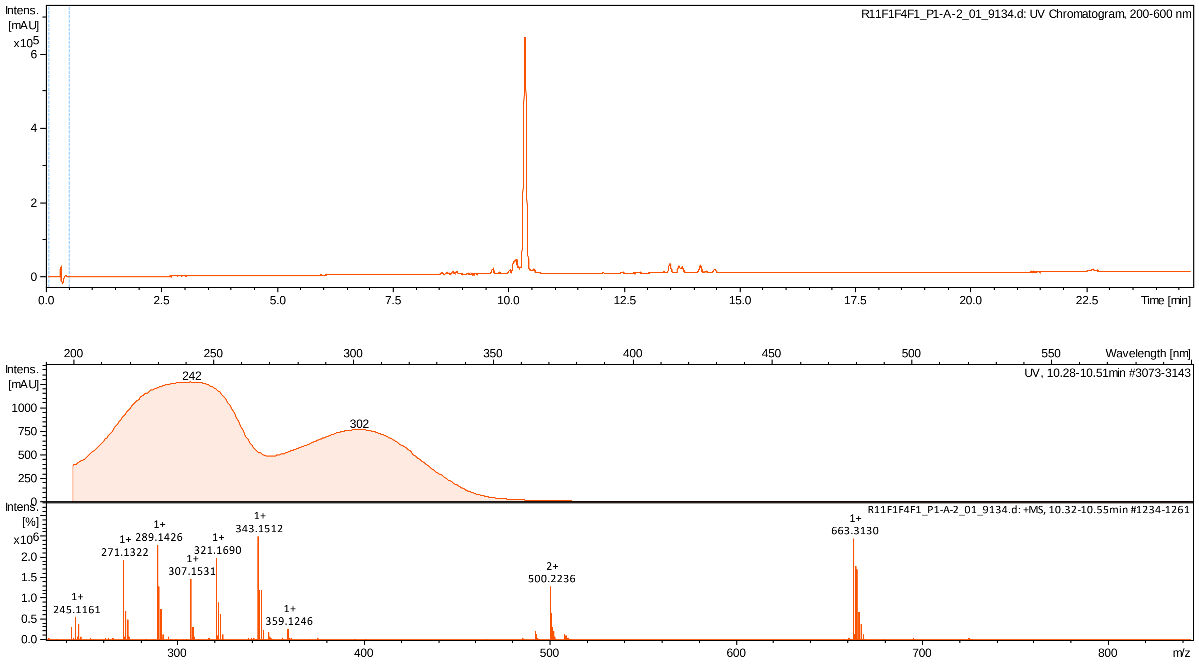

Figure S11. LC-MS chromatogram of compound **4**

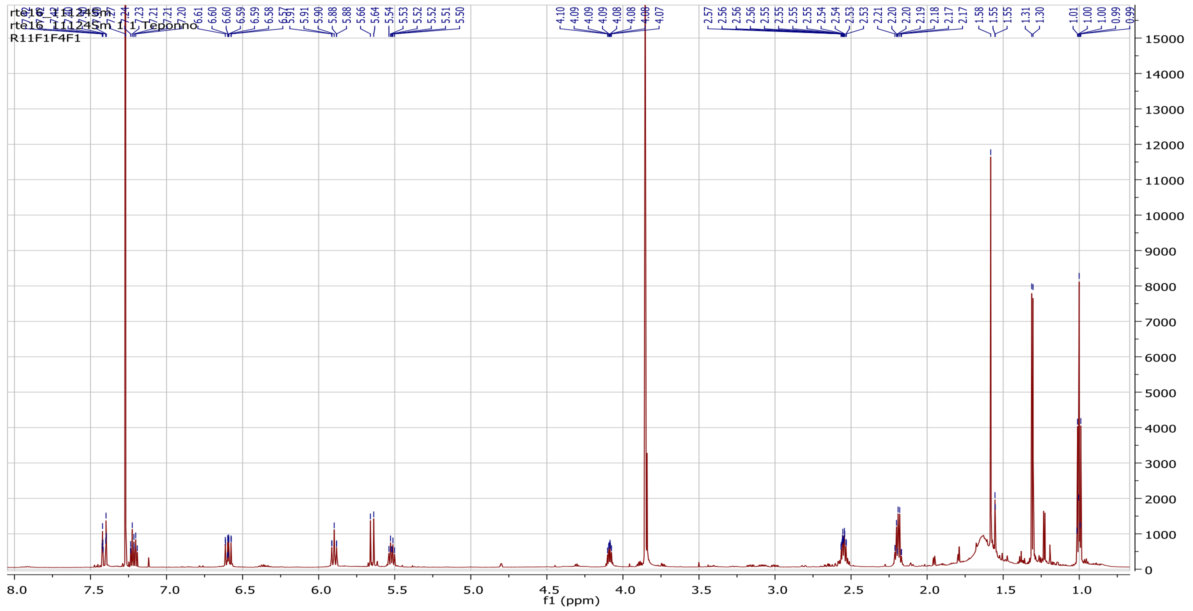

Figure S12. ^{1}H NMR spectrum of compound **4** (700 MHz, $CDCl_3$)

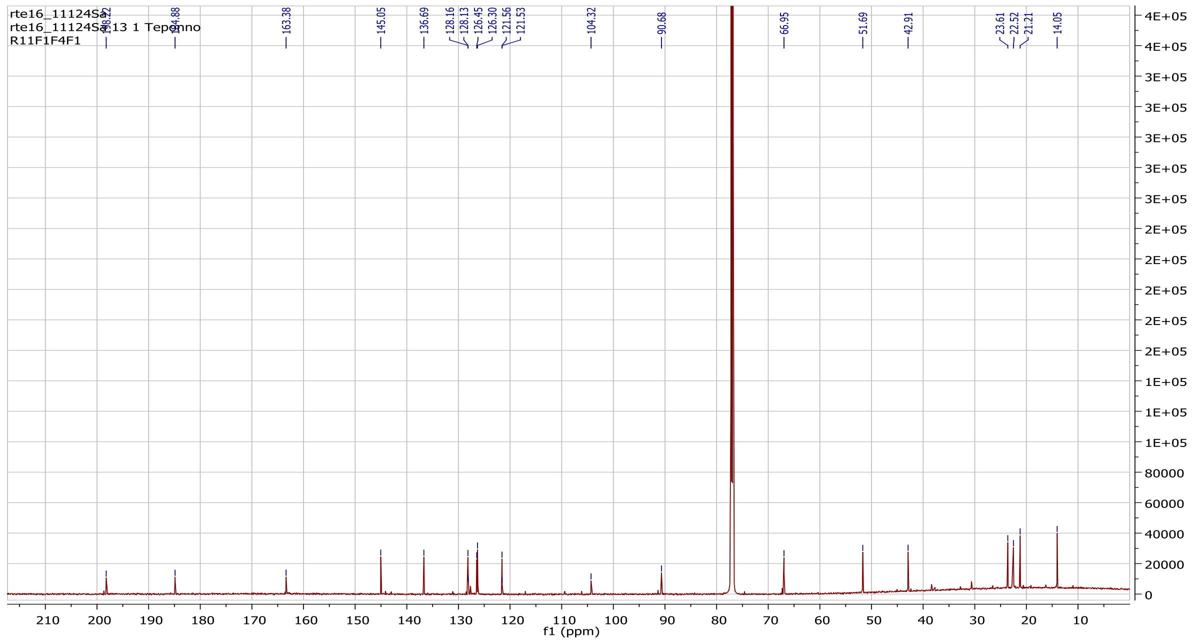

Figure S13. ^{13}C NMR spectrum of compound **4** (175 MHz, $CDCl_3$)

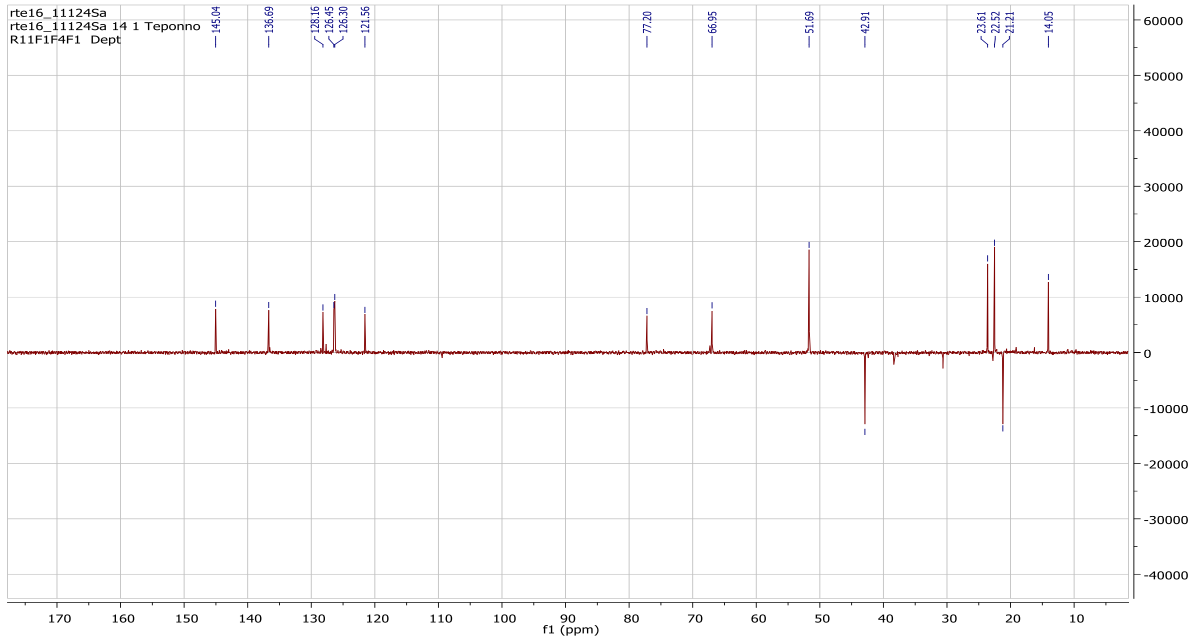

Figure S14. DEPT spectrum of compound **4** (175 MHz, $CDCl_3$)

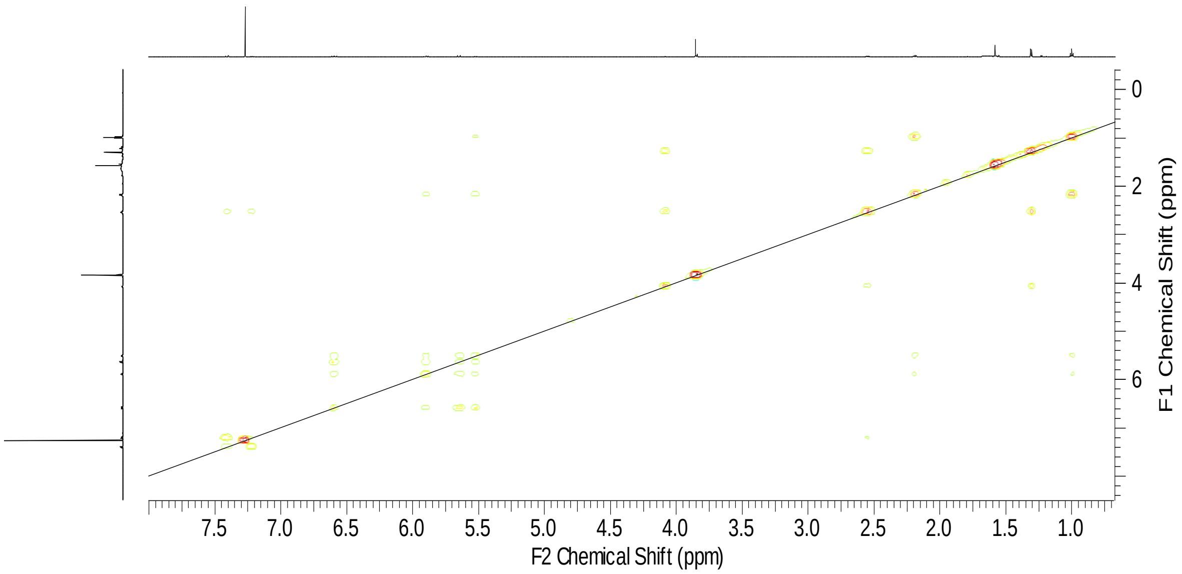

Figure S15. ^1H-^1H COSY spectrum of compound **4**

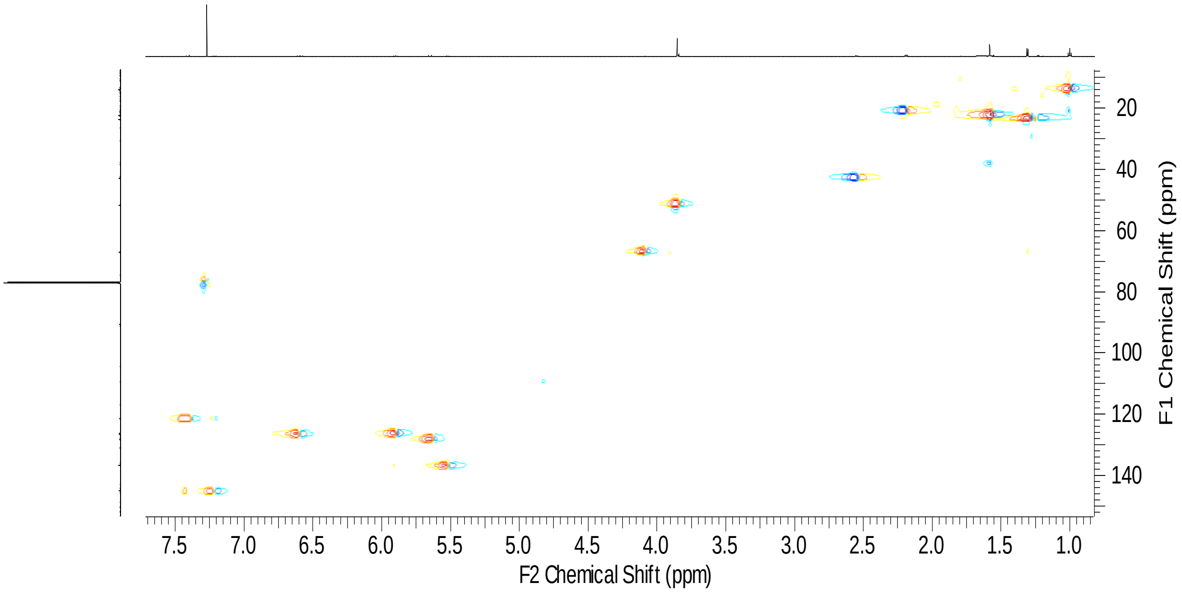

Figure S16. HSQC spectrum of compound **4**

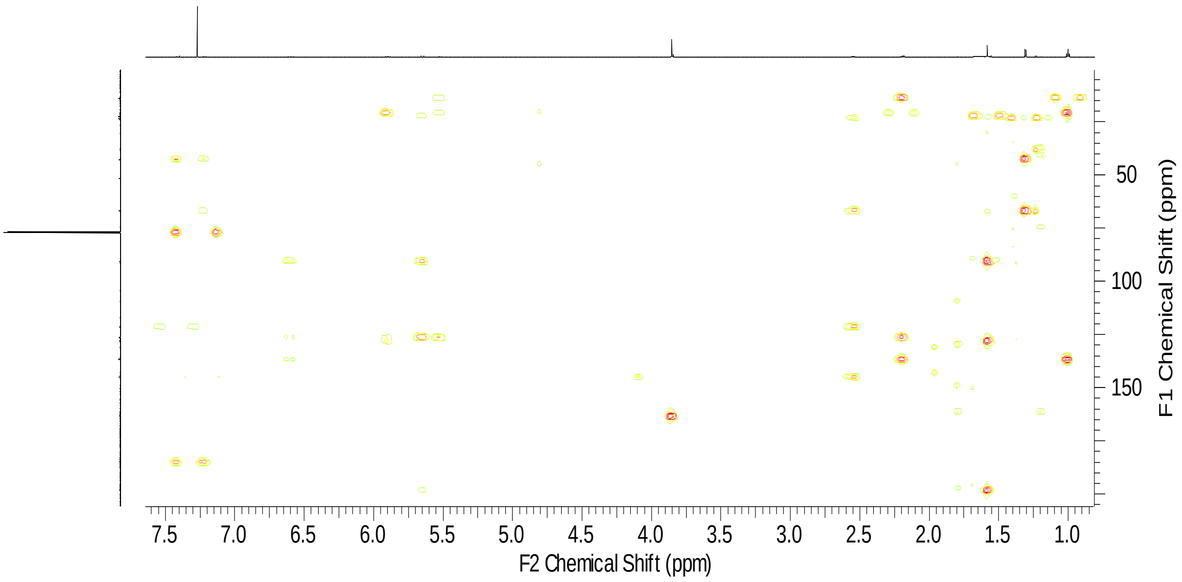

Figure S17. HMBC spectrum of compound **4**

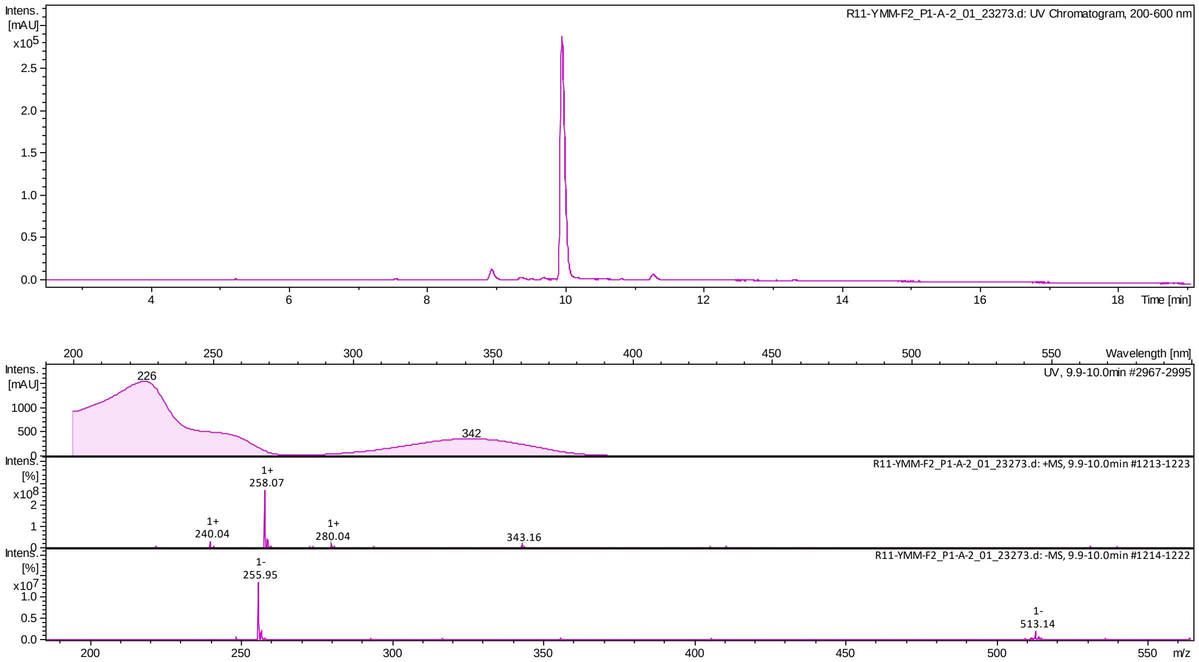

Figure S18. LC-MS chromatogram of compound **5**

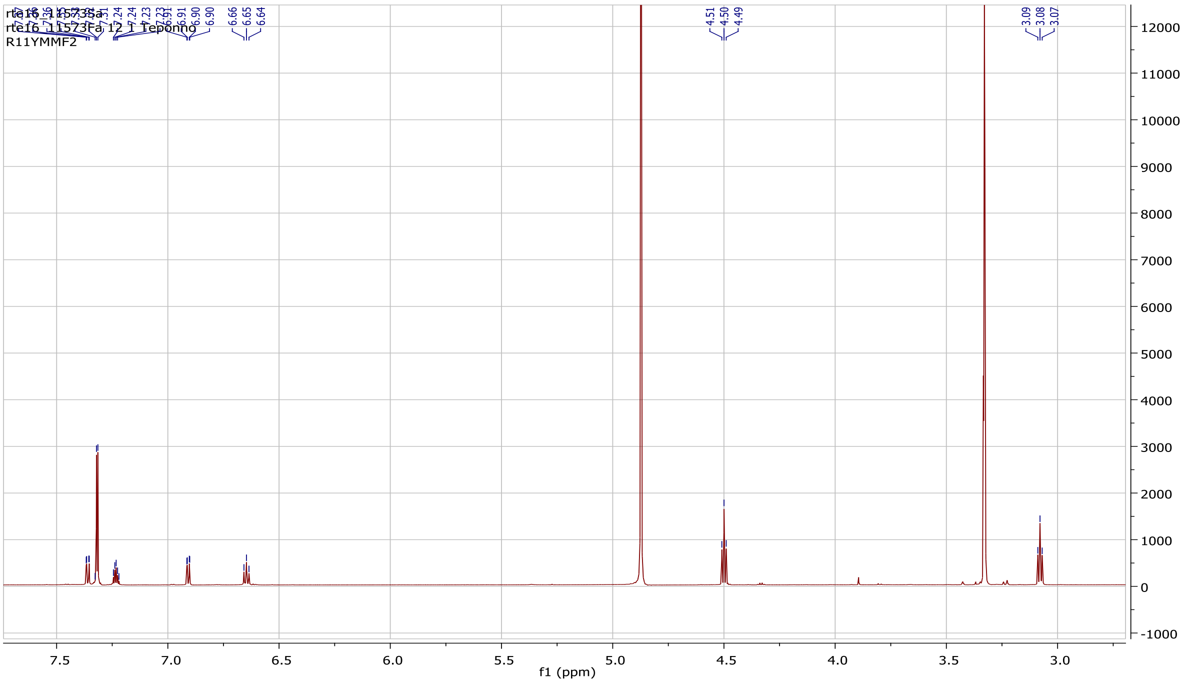

Figure S19. ^{1}H NMR spectrum of compound **5** (700 MHz, CD_3OD)

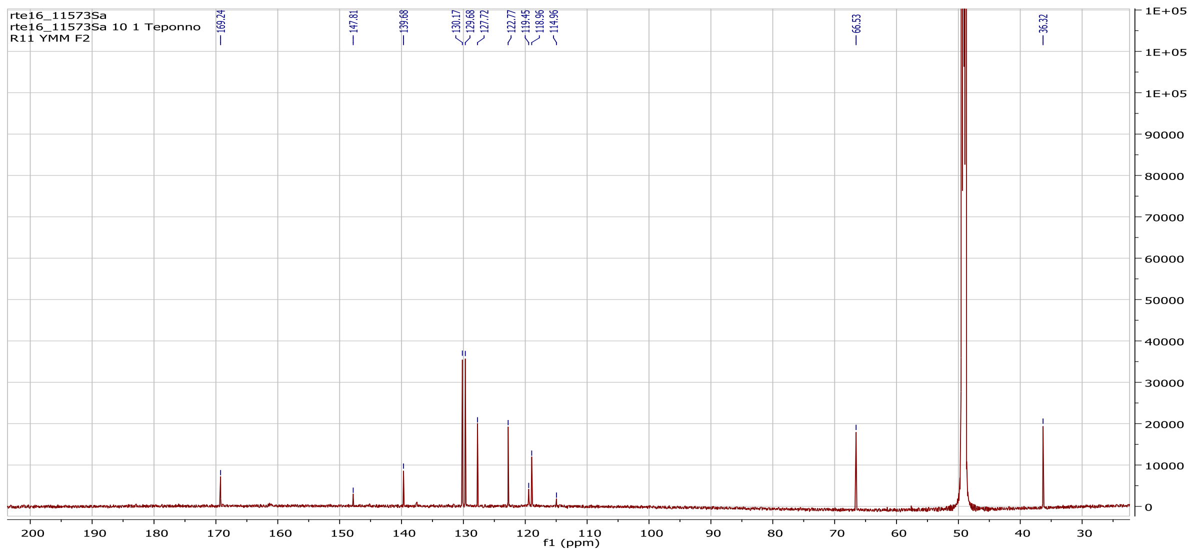

Figure S20. ^{13}C NMR spectrum of compound **5** (175 MHz, CD_3OD)

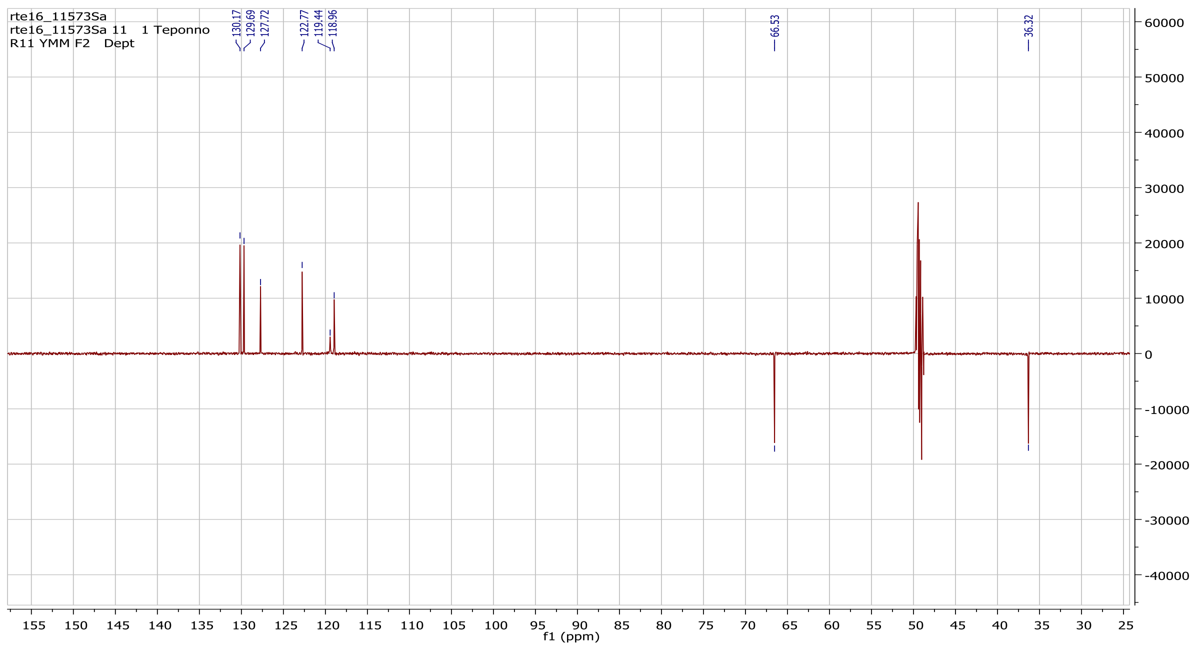

Figure S21. DEPT spectrum of compound **5** (175 MHz, CD_3OD)

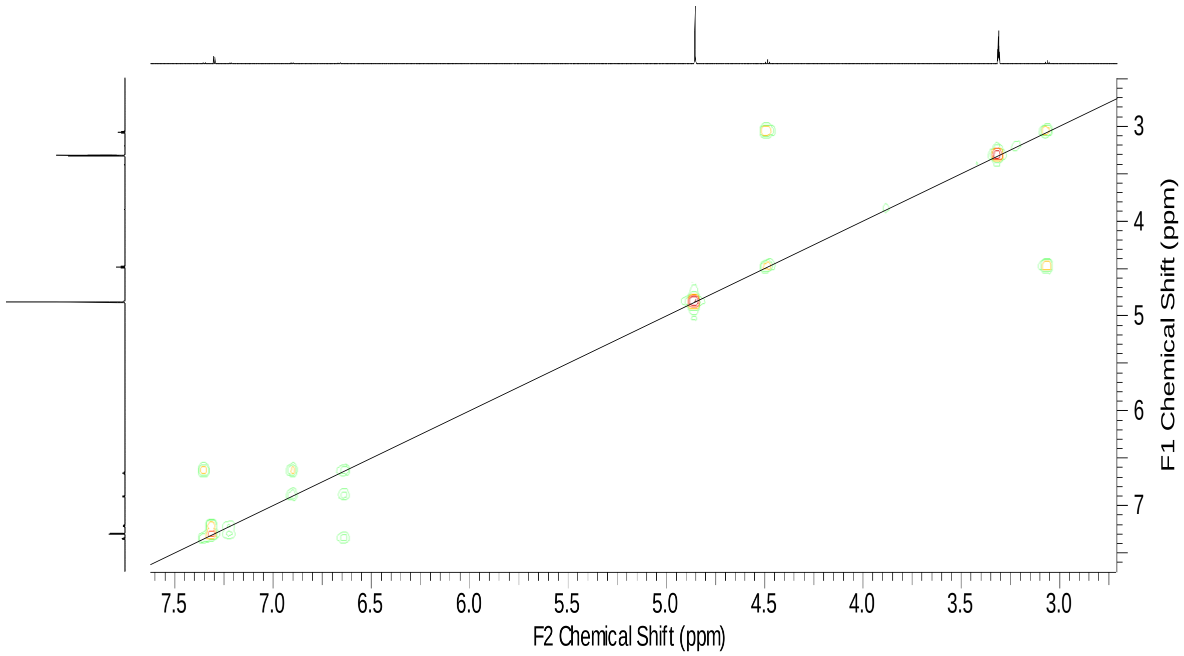

Figure S22. ^{1}H-^{1}H COSY spectrum of compound **5**

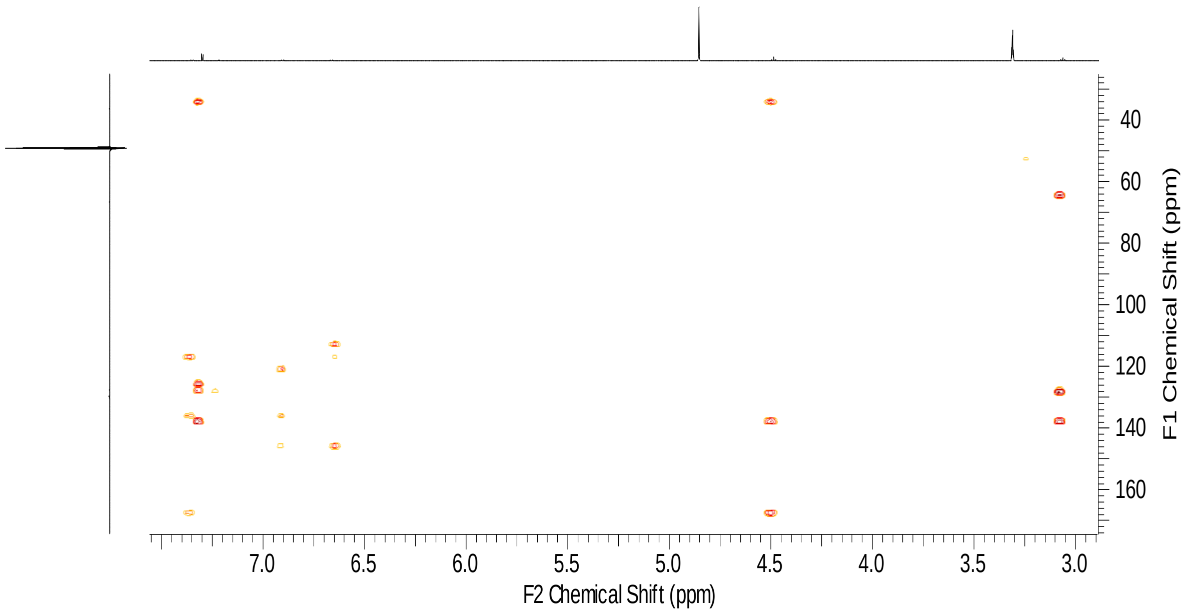

Figure S23. HMBC spectrum of compound **5**

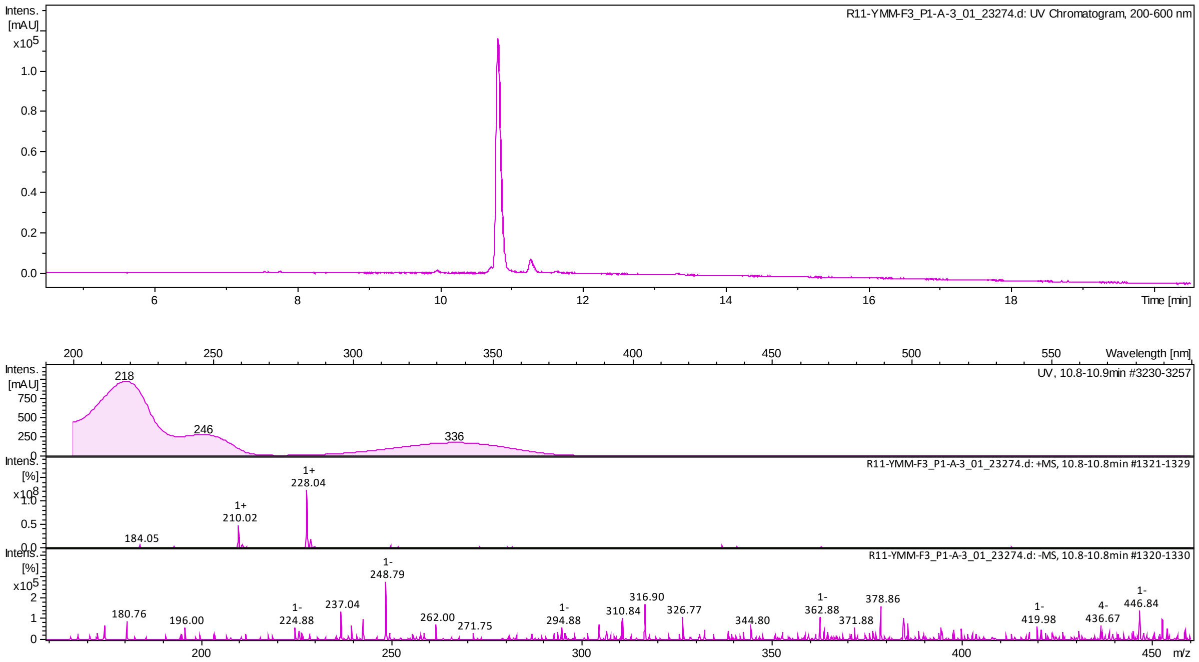

Figure S24. LC-MS chromatogram of compound **6**

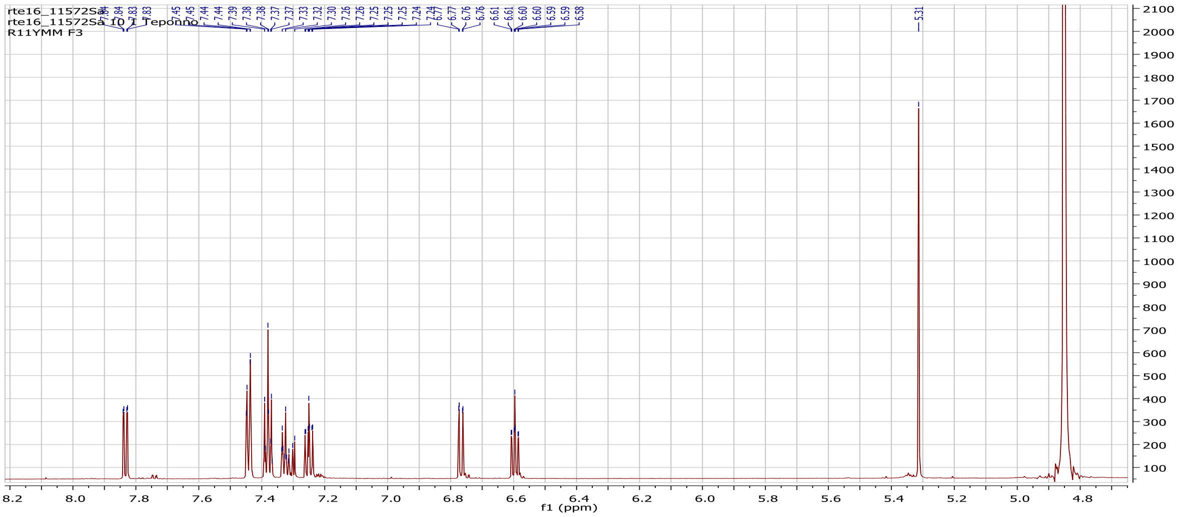

Figure S25. ^{1}H NMR spectrum of compound **6** (700 MHz, CD_3OD)

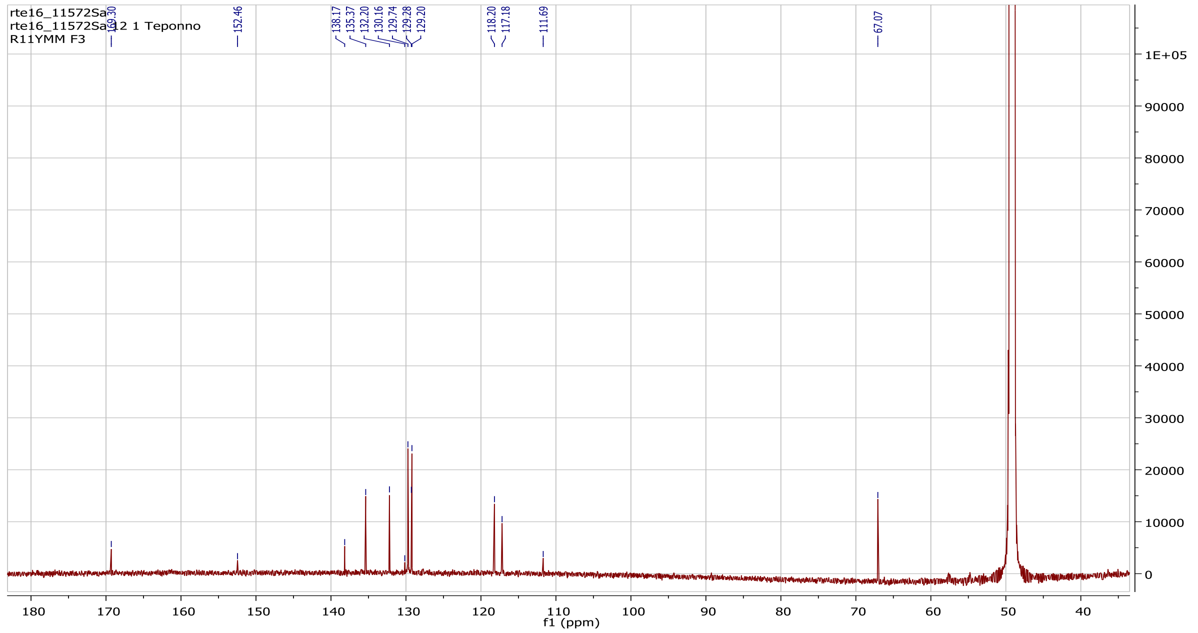

Figure S26. ^{13}C NMR spectrum of compound **6** (175 MHz, CD_3OD)

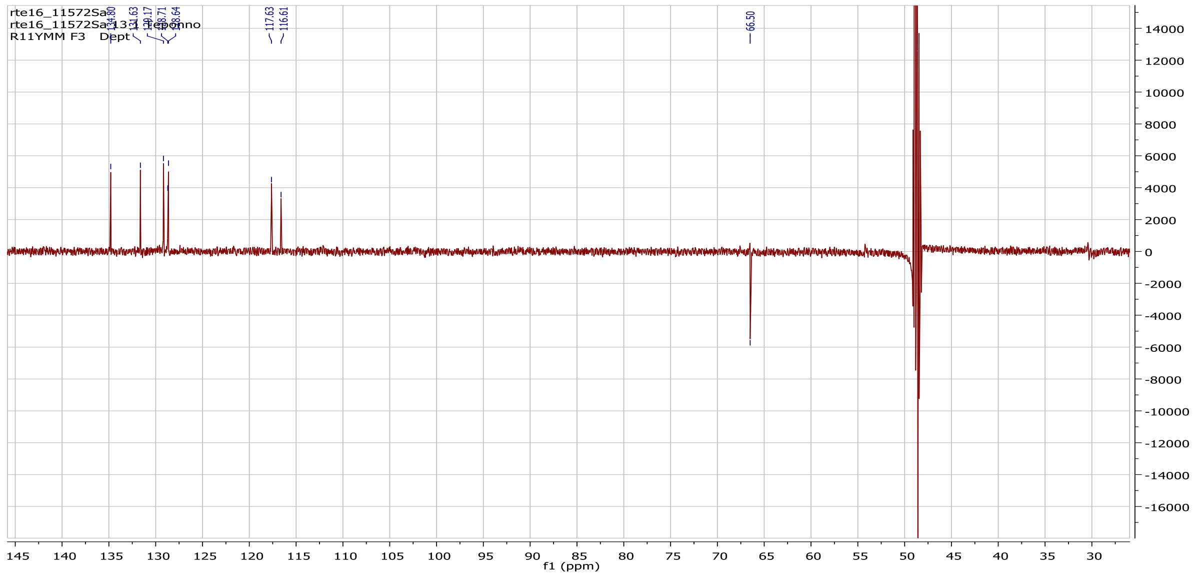

Figure S27. DEPT spectrum of compound **6** (175 MHz, CD_3OD)

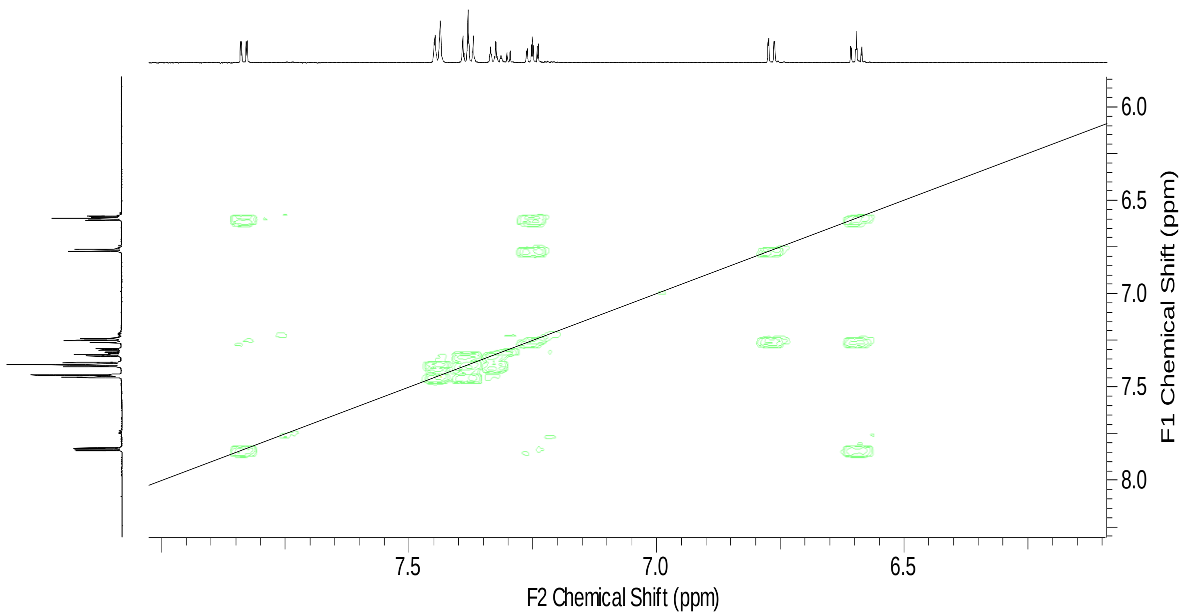

Figure S28. ^{1}H-^{1}H COSY spectrum of compound **6**

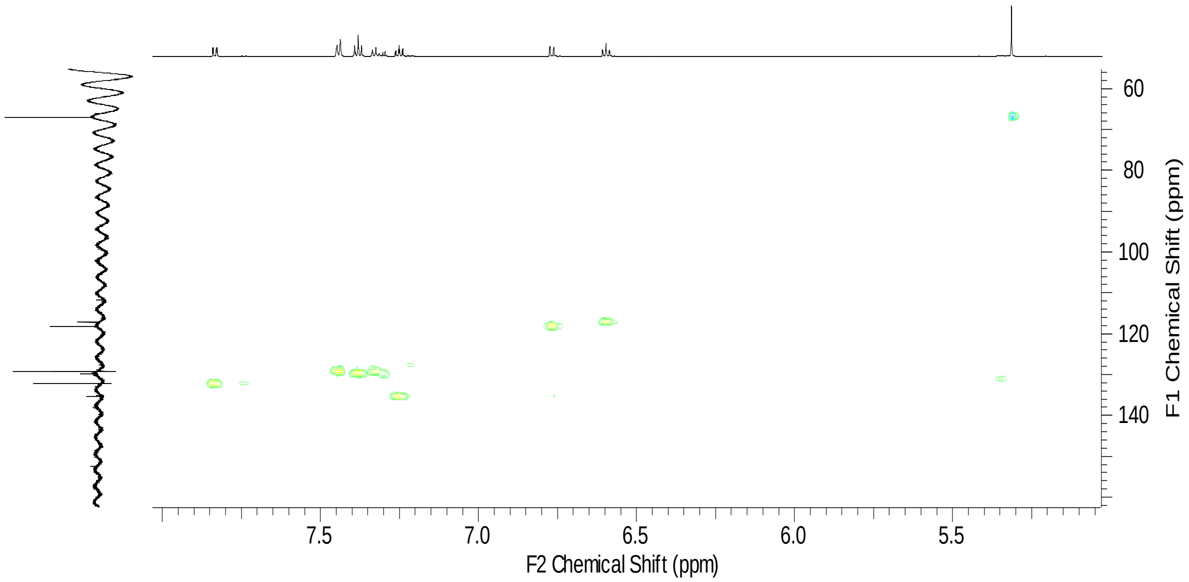

Figure S29. HSQC spectrum of compound **6**

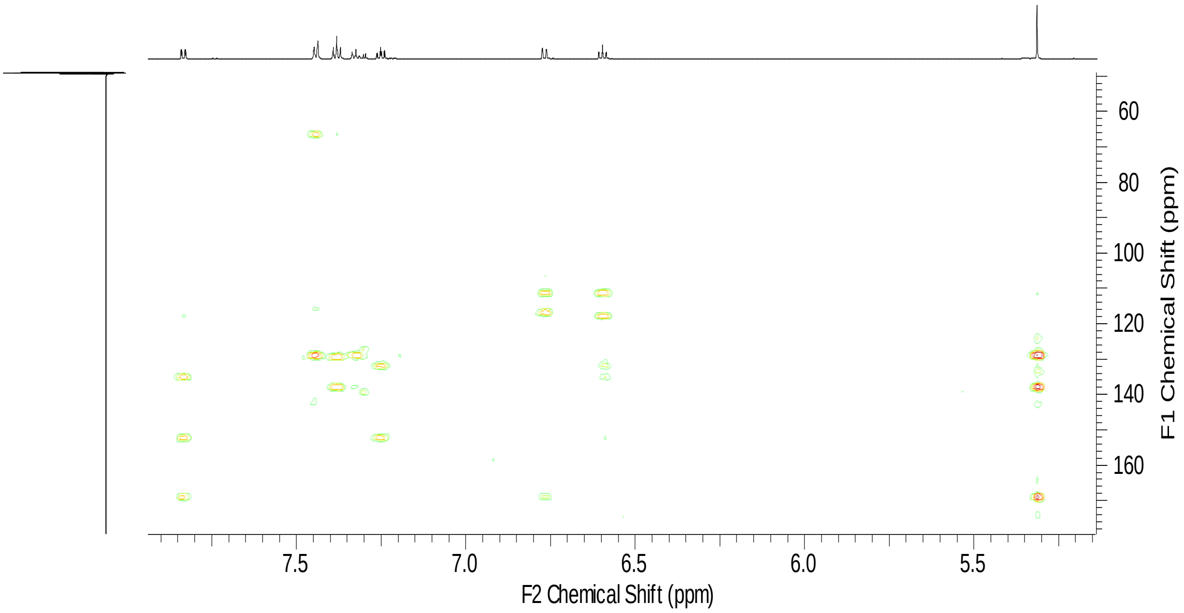

Figure S30. HMBC spectrum of compound **6**

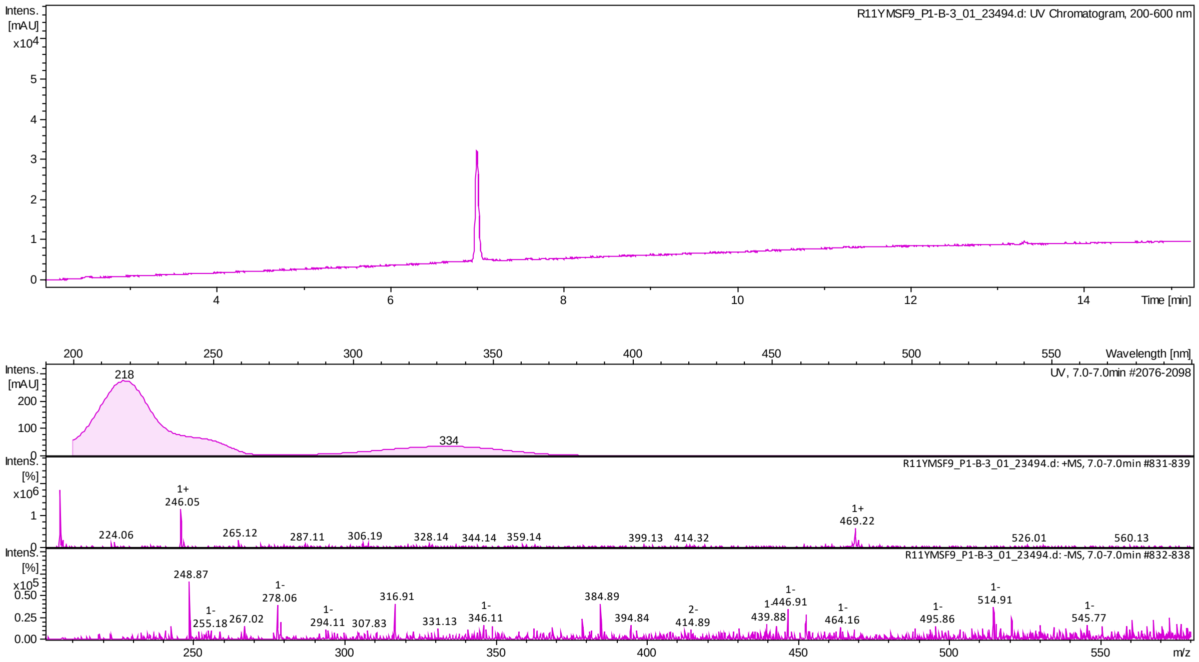

Figure S31. LC-MS chromatogram of compound **7**

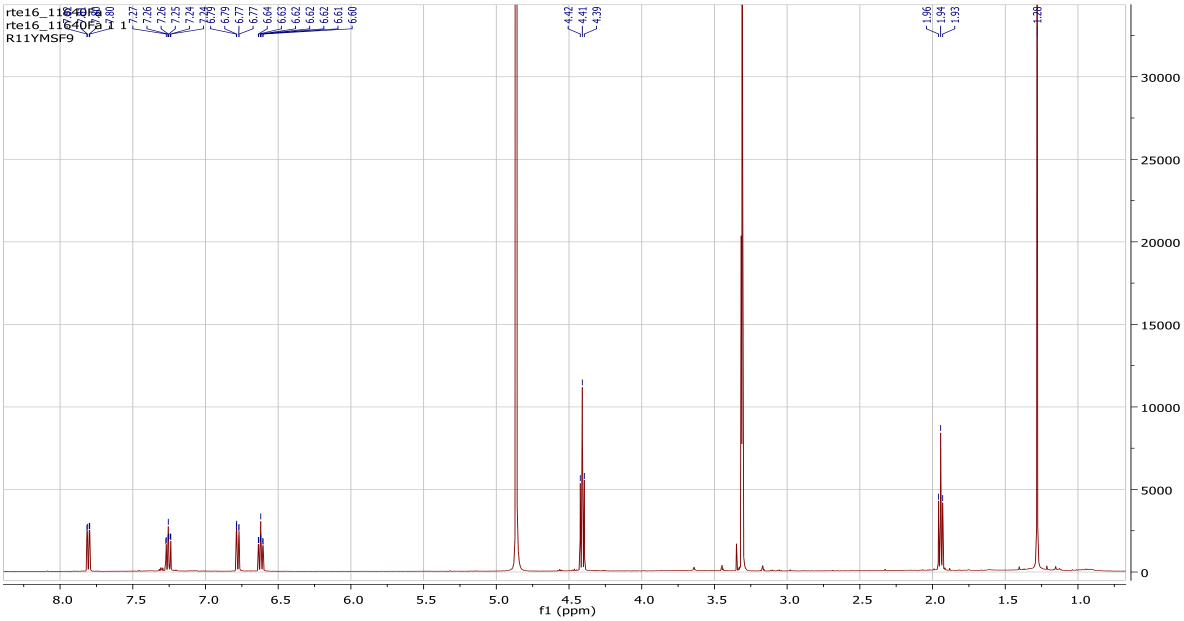

Figure S32. ^{1}H NMR spectrum of compound **7** (500 MHz, CD_3OD)

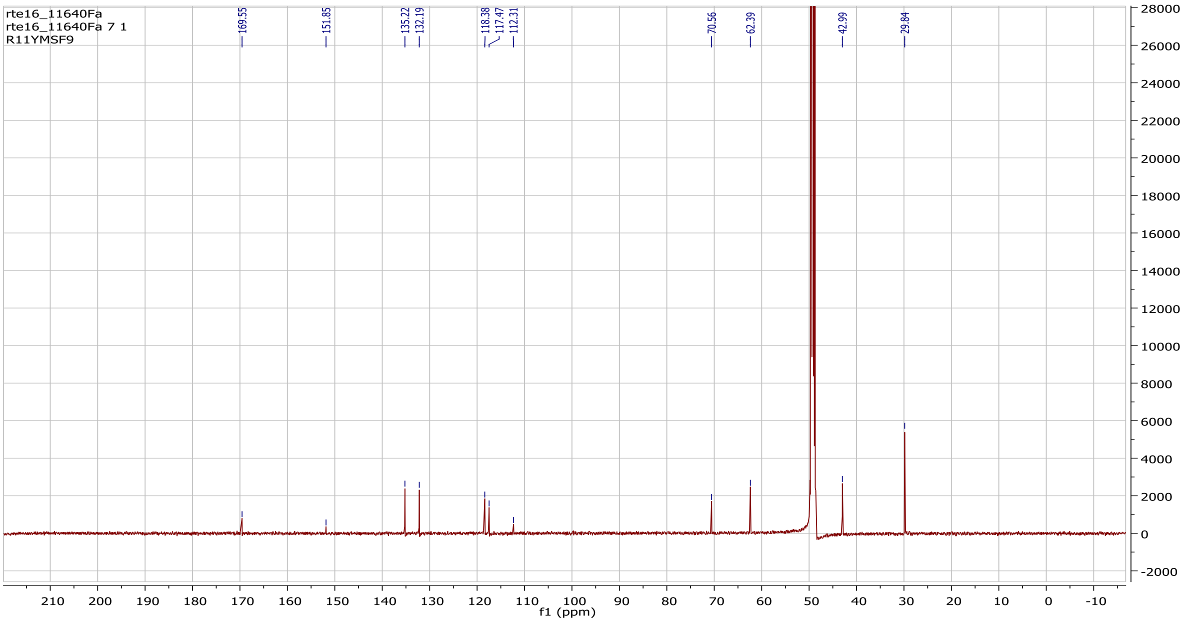

Figure S33. ^{13}C NMR spectrum of compound **7** (125 MHz, CD_3OD)

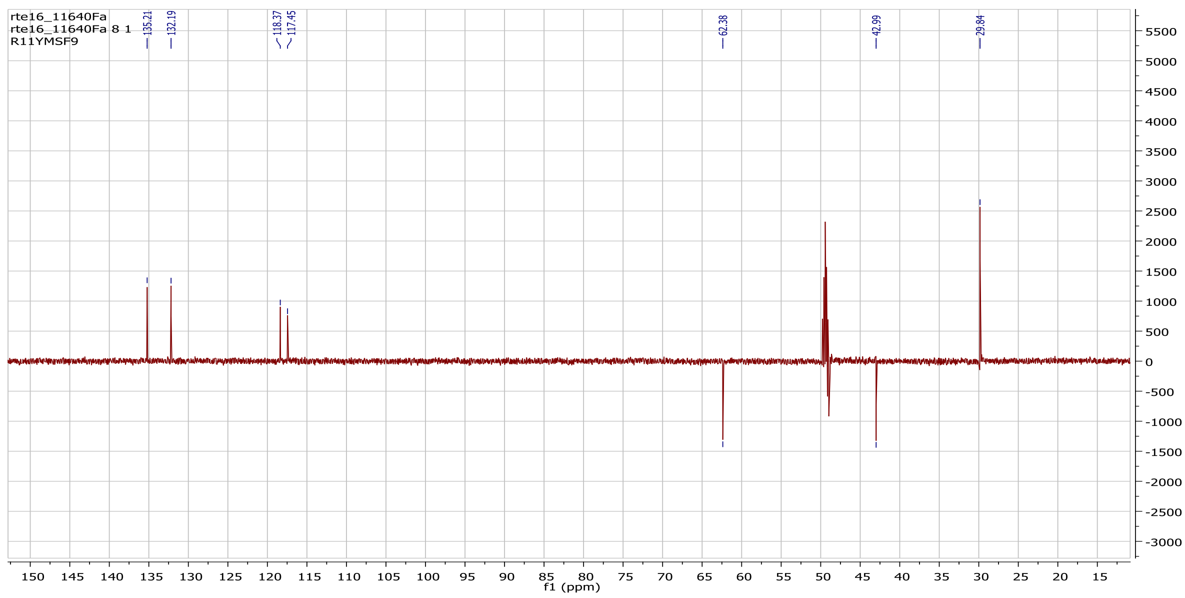

Figure S34. DEPT spectrum of compound **7** (125 MHz, CD_3OD)

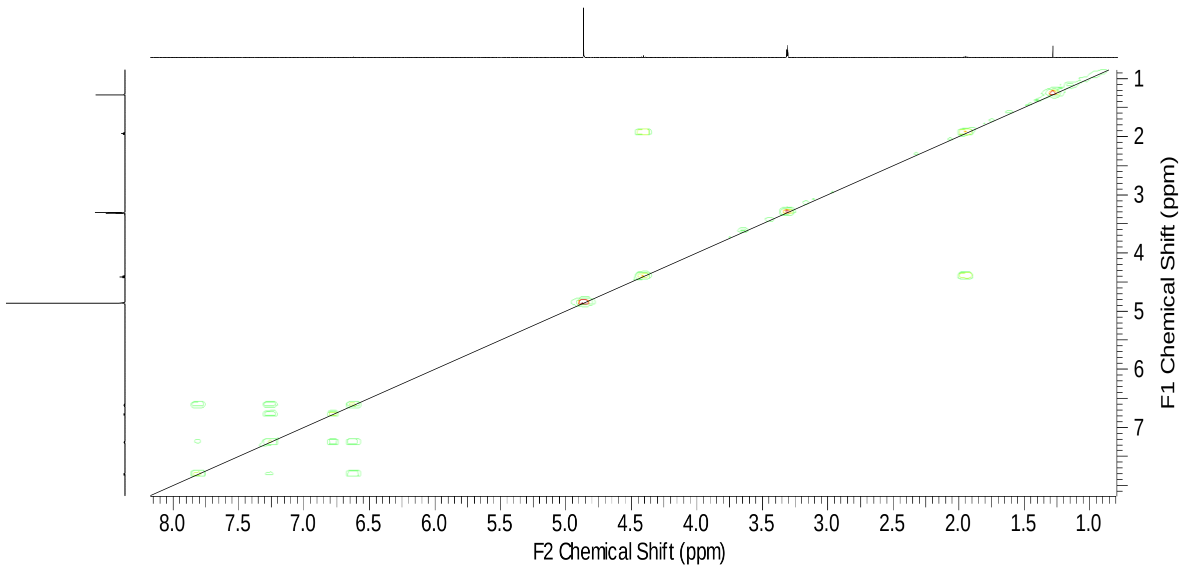

Figure S35. ^1H-^1H COSY spectrum of compound **7**

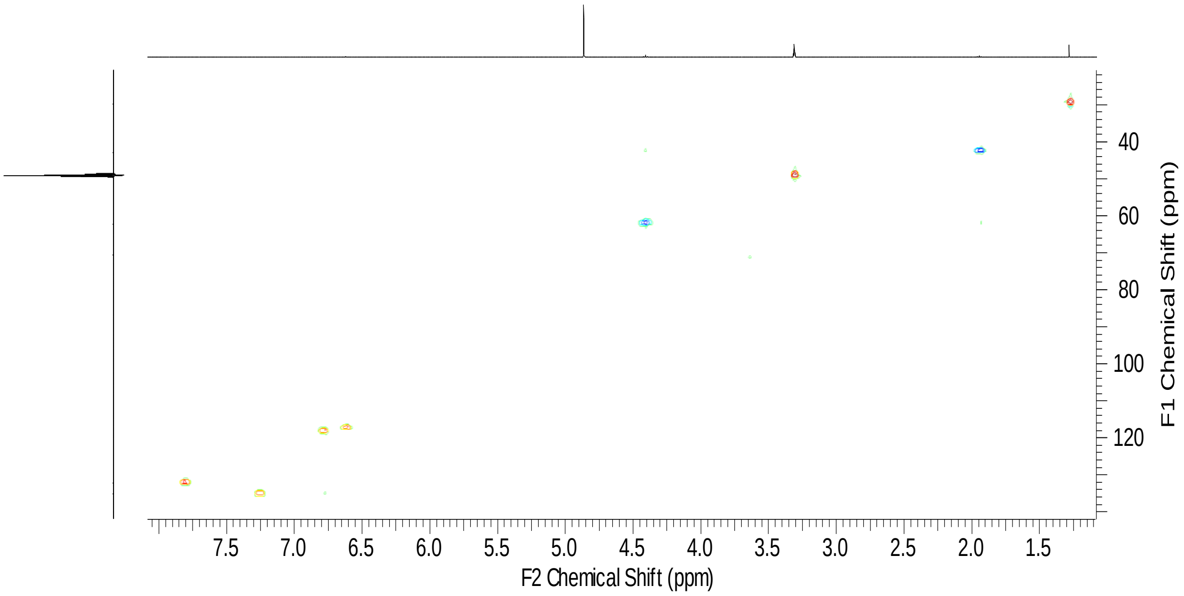

Figure S36. HSQC spectrum of compound **7**

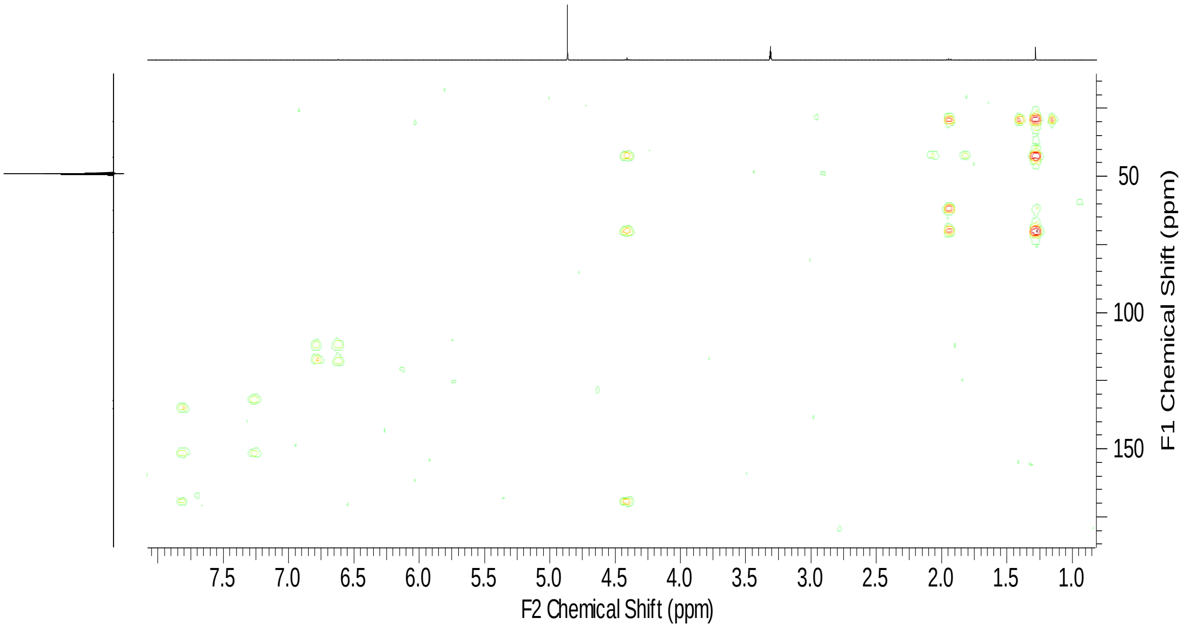

Figure S37. HMBC spectrum of compound **7**

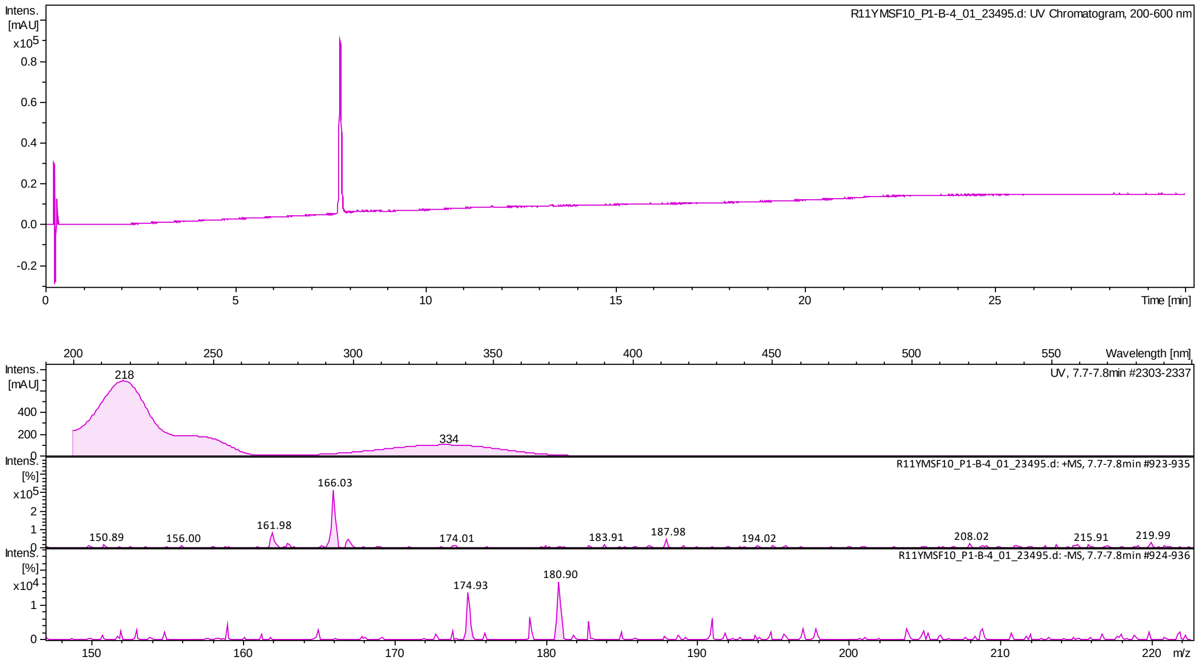

Figure S38. LC-MS chromatogram of compound **8**

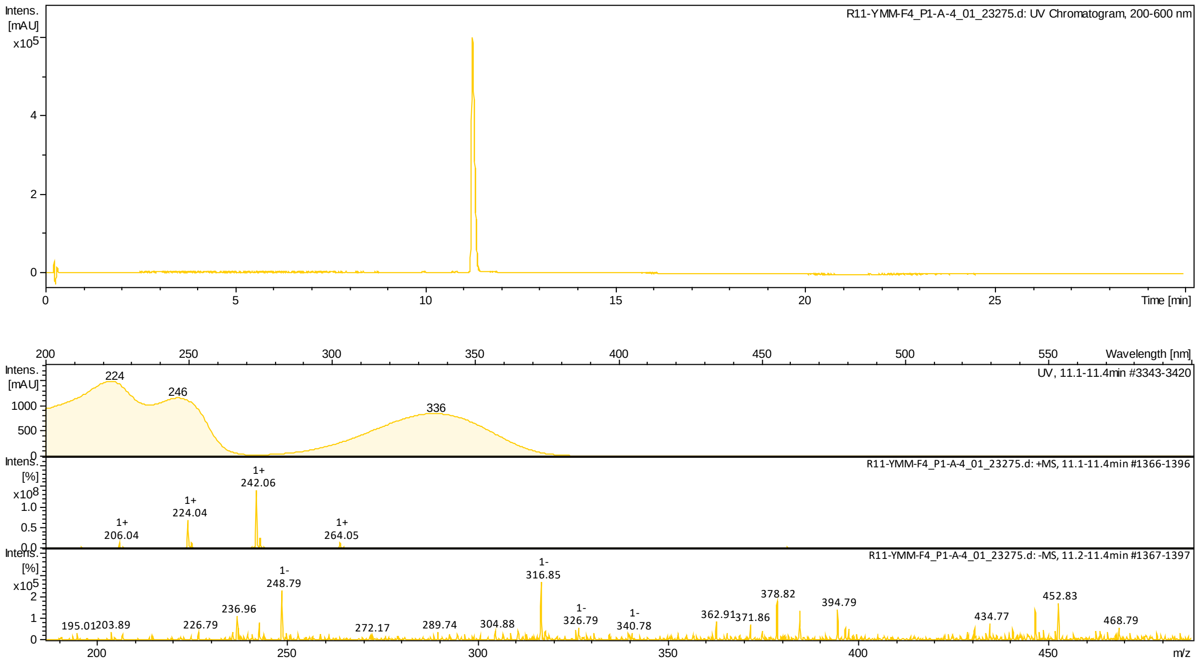

Figure S39. LC-MS chromatogram of compound **9**

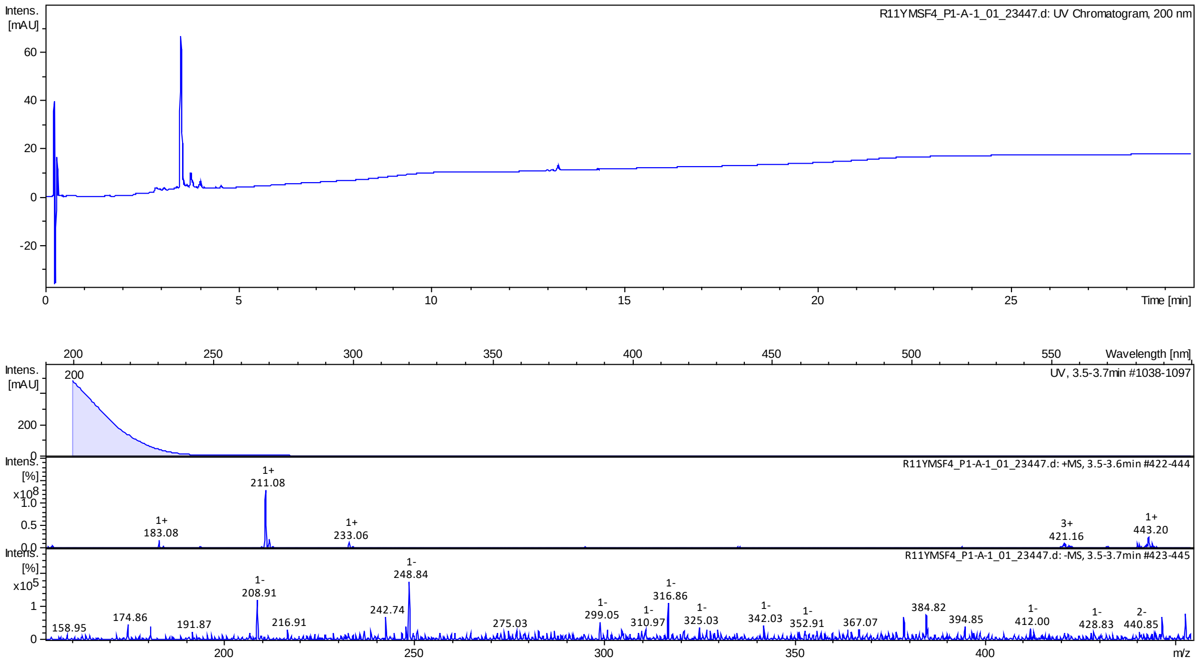

Figure S40. LC-MS chromatogram of compound **10**

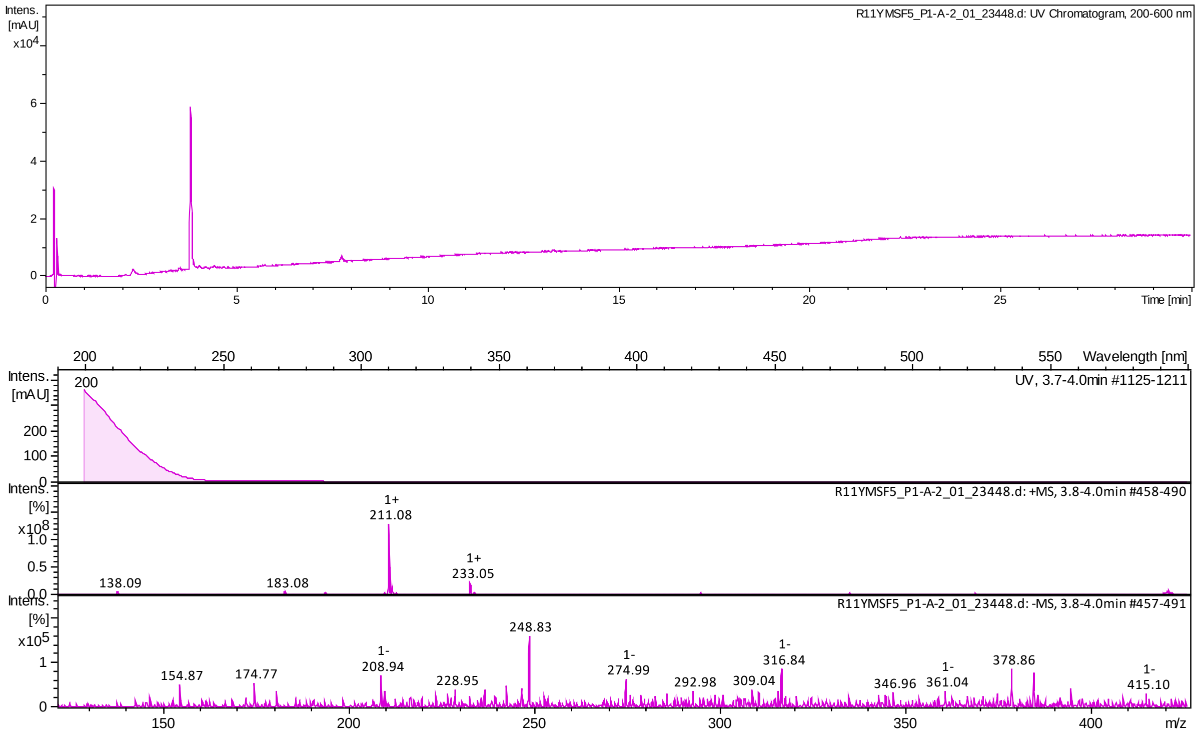

Figure S41. LC-MS chromatogram of compound **11**

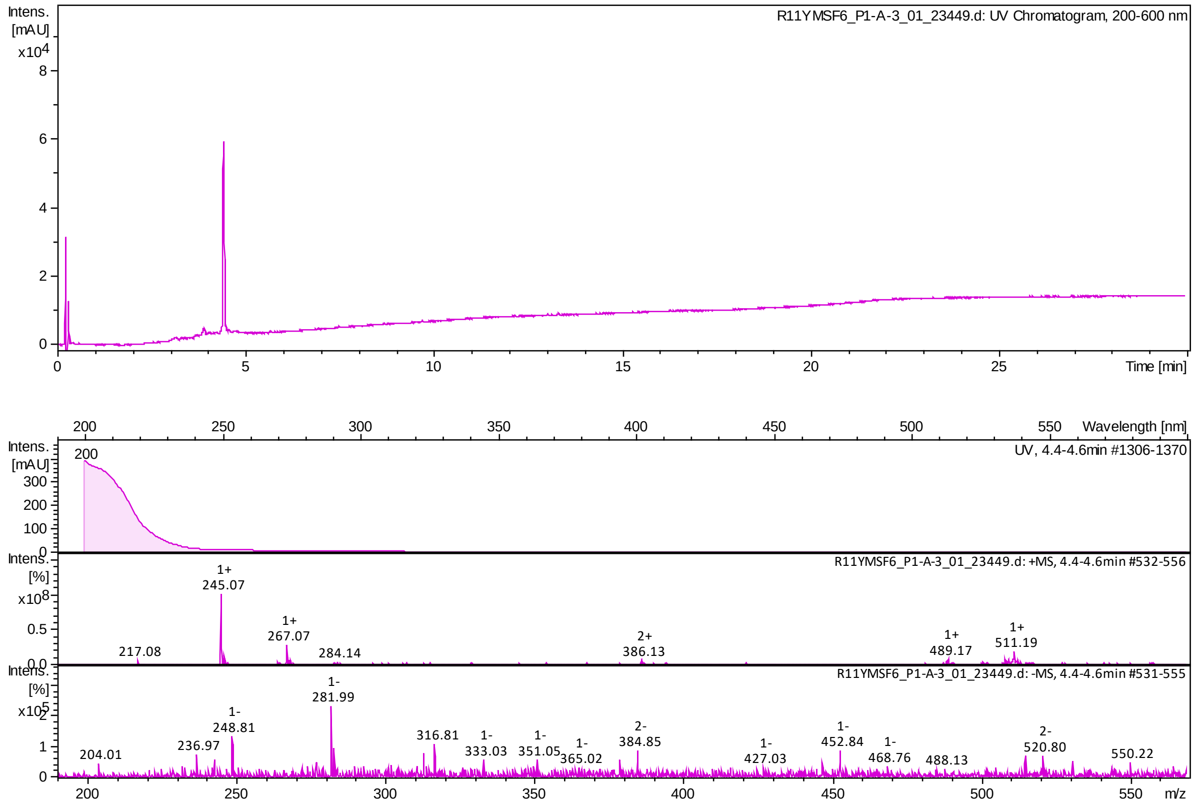

Figure S42. LC-MS chromatogram of compound **12**

Table S1. ^{1}H-NMR spectroscopic data for (*S*)- and (*R*)-MTPA esters of compound **1** (700 MHz, $CDCl_3$)

**Position**	**Compound 1:** δ_H **(** J **in Hz)**	***S*-Ester** δ_H **(** J **in Hz)**	***R*-Ester** δ_H **(** J **in Hz)**	Δ^{SR}
6′	1.00, t (7.3)	0.95, t (7.3)	0.95, t (7.3)	0.00
3″	2.55, m	2.70, m	2.65, m	+ 0.05
4″	4.08, m	4.27, m	4.37, m	– 0.10
5″	1.31, d (6.2)	1.34, d (6.2)	1.51, d (6.2)	– 0.17
Me-5	1.58, s	1.53, s	1.53, s	0.00

H-1″ and H-2″ are overlapped in the MTPA signals.

Table S2. ^{13}C- and ^{1}H-NMR Spectroscopic Data of Compounds 8 and 9 (CD_3OD, 125 and 500 MHz, resp.)

	**8**		**9**	
**Position**	δ_C,	δ_H	δ_C	δ_H
1	112.1	/	111.5	/
2	152.2	/	152.8	/
3	118.2	6.76, dd (8.3, 1.0)	117.9	6.72, dd (8.3, 1.0)
4	135.2	7.24, ddd (8.5, 7.1, 1.6)	135.2	7.21, m^b
5	117.2	6.60, ddd (8.2, 7.1, 1.1)	116.7	6.54, ddd (8.1, 7.1, 1.1)
6	132.2	7.80, dd (8.1, 1.6)	132.2	7.77, dd (8.1, 1.6)
7	169.6	/	169.6	/
1′	61.4	4.31, q (7.1)	139.8	/
2′	14.8	1.32, t (7.1)	130.2	7.30, m^b
3′			129.7	7.30, m^b
4′			127.7	7.21, m^b
5′			129.7	7.30, m^b
6′			130.2	7.30, m^b
7′			36.4	3.04, t (6.9)
8′			66.1	4.45, t (6.9)

[b]Overlapped

Table S3. MIC [µg/mL] values of compounds **1**, **2**, **4-12** sgainst the tested microorganisms.

**Test organism**	**MIC [µg/mL]**										
	**1**	**2**	**4**	**5**	**6**	**7**	**8**	**9**	**10**	**11**	**12**	**References**
Schizosaccharomyces pombe DSM 70572	n.a.	n.a.	n.a.	n.a.	n.a.	n.a.	n.a.	n.a.	n.a.	n.a.	n.a.	16.66[n]
Pichia anomala DSM 6766	n.a.	n.a.	n.a.	n.a.	n.a.	n.a.	n.a.	n.a.	n.a.	n.a.	n.a.	8.33[n]
Mucor hiemalis DSM 2656	n.a.	n.a.	n.a.	33.33	n.a.	n.a.	n.a.	16.66	n.a.	n.a.	n.a.	16.66[n]
Candida albicans DSM 1665	n.a.	n.a.	n.a.	n.a.	n.a.	n.a.	n.a.	n.a.	n.a.	n.a.	n.a.	16.66[n]
Rhodoturula glutinis DSM 10134	n.a.	n.a.	n.a.	66.67	n.a.	n.a.	n.a.	33.33	n.a.	n.a.	n.a.	2.08[n]
Micrococcus luteus DSM 1790	n.a.	n.a.	n.a.	16.66	n.a.	n.a.	n.a.	n.a.	n.a.	n.a.	n.a.	0.4[o]
Bacillus subtilis DSM 10	n.a.	n.a.	n.a.	8.33	n.a.	n.a.	n.a.	66.67	n.a.	n.a.	n.a.	4.16[o]
Escherichia coli DSM 1116	n.a.	n.a.	n.a.	n.a.	n.a.	n.a.	n.a.	n.a.	n.a.	n.a.	n.a.	3.33[o]
Staphylococcus aureus DSM 346	n.a.	n.a.	n.a.	66.67	n.a.	n.a.	n.a.	66.67	n.a.	n.a.	n.a.	0.1[o]
Mycobacterium smegmatis DSM ATCC 700084	n.a.	n.a.	n.a.	n.a.	n.a.	n.a.	n.a.	n.a.	n.a.	n.a.	n.a.	2.08[k]
Chromobacterium violaceum DSM 30191	n.a.	n.a.	n.a.	n.a.	n.a.	n.a.	n.a.	n.a.	n.a.	n.a.	n.a.	0.4[o]
Pseudomonas aeruginosa DSM PA14	n.a.	n.a.	n.a.	n.a.	n.a.	n.a.	n.a.	n.a.	n.a.	n.a.	n.a.	0.52[g]

n.a.: No Activity, [n] Nystatin 1 mg/ml, [o] Oxytetracyclin 1 mg/ml, [k] Kanamycin 10 mg/ml, [g] Gentamycin 1 mg/ml

Table S4. Cytotoxic effect (IC_{50}) of compounds **1**, **2**, **4-12** against two cancer cell lines

	IC_{50} (*μ*g/mL)											IC_{50} (*n*g/mL)
**Cell line**	**1**	**2**	**4**	**5**	**6**	**7**	**8**	**9**	**10**	**11**	**12**	**Epothilone B**
KB3.1	n.a.	n.a.	18	18	n.a.	n.a.	n.a.	8.8	n.a.	n.a.	n.a.	0.052
L929	n.a.	n.a.	19	no	n.a.	n.a.	n.a.	no	n.a.	n.a.	n.a.	1.4

na: Not active; *no*: IC_{50} not obtained

References

1. Verkley, G.J.M.; Dukik, K.; Renfurm, R.; Göker, M.; Stielow, J.B. Novel genera and species of the coniothyrium-like fungi in Montagnulaceae (Ascomycota). *Persoonia Mol. Phylogeny Evol. Fungi* **2014**, *32*, 25–51.
